# Anomaly detection in mixed high-dimensional molecular data

**DOI:** 10.1093/bioinformatics/btad501

**Published:** 2023-08-16

**Authors:** Lena Buck, Tobias Schmidt, Maren Feist, Philipp Schwarzfischer, Dieter Kube, Peter J Oefner, Helena U Zacharias, Michael Altenbuchinger, Katja Dettmer, Wolfram Gronwald, Rainer Spang

**Affiliations:** Department of Statistical Bioinformatics, University of Regensburg, 93040 Regensburg, Germany; Department of Statistical Bioinformatics, University of Regensburg, 93040 Regensburg, Germany; Department of Hematology and Medical Oncology, University Medicine Gottingen, 37075 Gottingen, Germany; Institute of Functional Genomics, University of Regensburg, 93040 Regensburg, Germany; Department of Hematology and Medical Oncology, University Medicine Gottingen, 37075 Gottingen, Germany; Institute of Functional Genomics, University of Regensburg, 93040 Regensburg, Germany; Peter L. Reichertz Institute for Medical Informatics of TU Braunschweig and Hannover Medical School, Hannover Medical School, 30625 Hannover, Germany; Department of Medical Bioinformatics, University Medical Center Göttingen, 37075 Göttingen, Germany; Institute of Functional Genomics, University of Regensburg, 93040 Regensburg, Germany; Institute of Functional Genomics, University of Regensburg, 93040 Regensburg, Germany; Department of Statistical Bioinformatics, University of Regensburg, 93040 Regensburg, Germany

## Abstract

**Motivation:**

Mixed molecular data combines continuous and categorical features of the same samples, such as OMICS profiles with genotypes, diagnoses, or patient sex. Like all high-dimensional molecular data, it is prone to incorrect values that can stem from various sources for example the technical limitations of the measurement devices, errors in the sample preparation, or contamination. Most anomaly detection algorithms identify complete samples as outliers or anomalies. However, in most cases, not all measurements of those samples are erroneous but only a few one-dimensional features within the samples are incorrect. These one-dimensional data errors are continuous measurements that are either located outside or inside the normal ranges of their features but in both cases show atypical values given all other continuous and categorical features in the sample. Additionally, categorical anomalies can occur for example when the genotype or diagnosis was submitted wrongly.

**Results:**

We introduce ADMIRE (Anomaly Detection using MIxed gRaphical modEls), a novel approach for the detection and correction of anomalies in mixed high-dimensional data. Hereby, we focus on the detection of single (one-dimensional) data errors in the categorical and continuous features of a sample. For that the joint distribution of continuous and categorical features is learned by mixed graphical models, anomalies are detected by the difference between measured and model-based estimations and are corrected using imputation. We evaluated ADMIRE in simulation and by screening for anomalies in one of our own metabolic datasets. In simulation experiments, ADMIRE outperformed the state-of-the-art methods of Local Outlier Factor, stray, and Isolation Forest.

**Availability and implementation:**

All data and code is available at https://github.com/spang-lab/adadmire. ADMIRE is implemented in a Python package called adadmire which can be found at https://pypi.org/project/adadmire.

## 1 Introduction

Molecular data are error-prone. Systematic errors in sample collection or preparation can affect large sets of features and need to be corrected using normalization methods. Additionally, technical problems can affect individual measurements. Due to the different molecular properties of the measured features, it is often the case that a sample shows only in a few of its measured features abnormalities while the rest of them are inconspicuous. Also, not all samples might be affected in the same way as each sample is usually processed separately and therefore is exposed to a different kind of error source. Consequently, molecular datasets contain individual data errors that can affect each measured feature in each sample in a different way. These one-dimensional data errors are especially hard to detect in the setting of high-dimensional molecular datasets. Furthermore, they might present themselves as univariate outliers, with measured values exceeding the range of the features by multiple orders. But they also appear as anomalies when a value fits well into the univariate distribution of its feature, but not into the joint distribution of all features. For example, if a gene shows expression values between 4–6 in men and between 8–14 in women, a value of 12 in a man is suspicious.

More formally, a given value *x_ij_* of a feature *j* in a sample *i* might be a typical value for the marginal distribution of feature *j*, but not for its conditional distribution given all other features of sample *i*. These anomalies can only be detected when the information given by the categorical, phenotypic information is taken into consideration as well. But this data can also contain anomalies. Data entry errors or a mix-up during the experimental procedure can lead to artifacts in the phenotypical information of a dataset. Samples are then assigned, e.g. to the wrong treatment class, a female participant is considered as a male, etc.

The literature knows numerous methods for detecting univariate outliers in molecular data (Grubbs 1969) and for detecting multivariate anomalies in continuous (Korn *et al.* 2001, DeCoste and Levine 2004, Hodge and Austin 2004, Ando 2007) as well as in discrete data (John 1995). A common approach to anomaly detection is using the *k*-nearest neighbors to detect anomalies within this neighborhood as done by the Local Outlier Factor (LOF) (Breunig *et al.* 2000) and the Search and TRace AnomalY (stray) algorithm (Talagala *et al.* 2021), or to use random forests to isolate anomalous samples (see Isolation Forest; Liu *et al.* 2008). Unlike our method which aims at the detection of anomalies in individual entries of the data matrix, those algorithms however confine themselves to identifying suspicious samples, see Supplementary data.

Most datasets in molecular biology are mixed. Continuous OMICS data are complemented by discrete phenodata-like patient characteristics (sex, diagnosis, treatment), experimental conditions (experimental groups, controls), or technical designs (batches, repetitions). Therefore, we developed a novel approach to anomaly detection based on mixed graphical models (MGMs). MGMs (Lee and Hastie 2015, Cheng *et al.* 2017) are well-established generalizations of Gaussian graphical models (GGMs) (Lauritzen 1996, Meinshausen and Bühlmann 2006) to mixed data. Beyond anomaly detection MGMs have been successfully used for studying the structure of metabolic, proteomic, or transcriptomic networks (Chun *et al.* 2013, Wang *et al.* 2016, Zhao and Duan 2019, Altenbuchinger *et al.* 2020). We briefly review the concept of MGMs, describe how ADMIRE (Anomaly Detection using MIxed gRaphical modEls) detects anomalies, handles missing values, validate it in simulation experiments, compare it to alternative approaches and demonstrate its power in the contexts of finding experimental artefacts in a state-of-the-art metabolomics dataset.

## 2 Materials and methods

In a nutshell, ADMIRE fits for each sample in a leave-one-out approach a MGM to the mixed dataset. From this MGM, we derive the conditional distribution of a feature given all other features. We then compare an actual observation of a specific feature in a specific sample with its corresponding conditional distribution. If the value is far away from what can be expected from the model given all other features of the same sample, we flag it as anomaly and the user may choose to replace it by a model-based imputation.

### 2.1 Mixed graphical models

**Figure 1.**
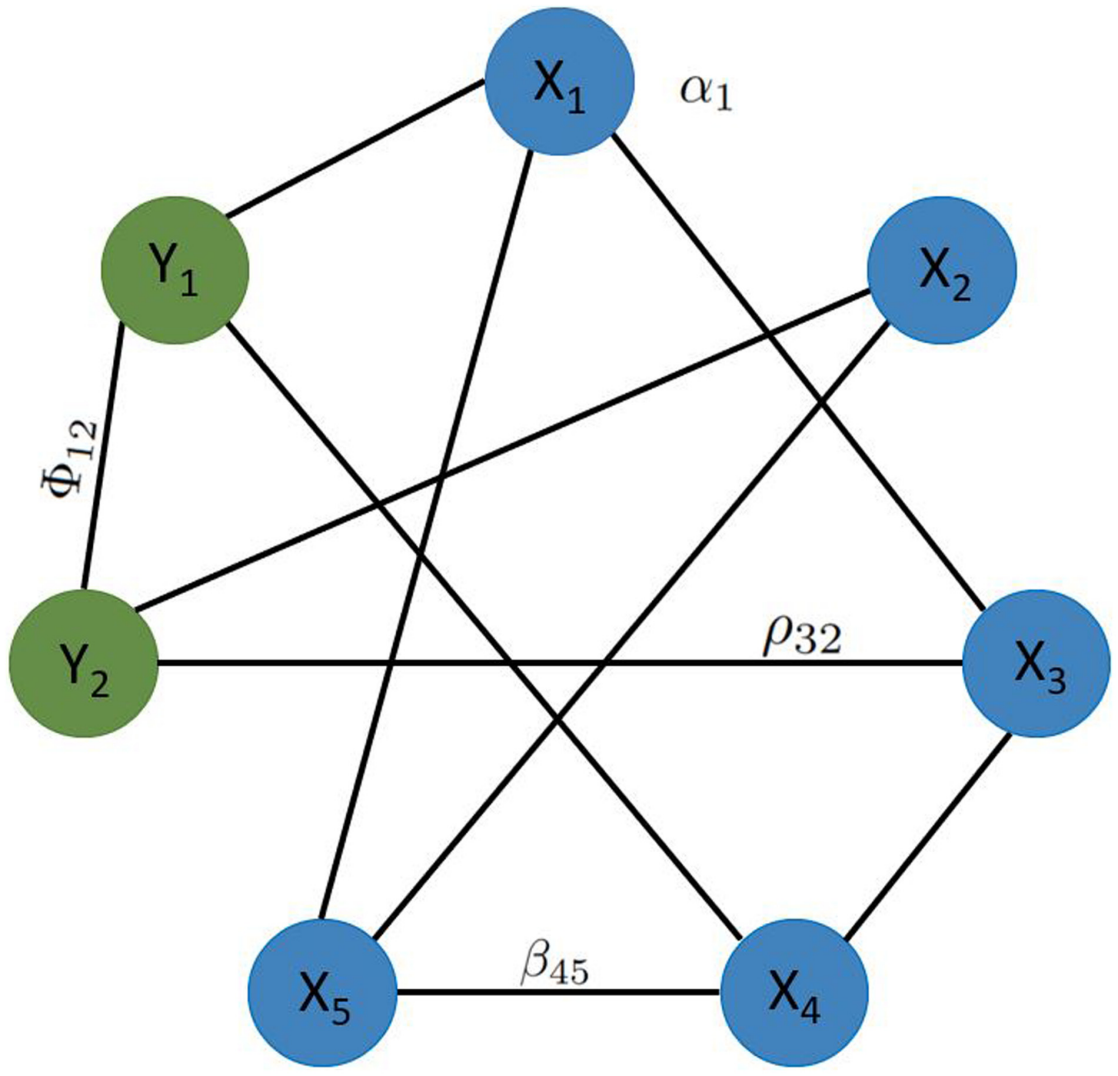
A mixed graphical model. The nodes include both continuous features ($X_{1},\;...,\;X_{5}$) and discrete features ($Y_{1}$and $Y_{2}$). A missing edge between two nodes denotes their conditional independence given all other variables. The node and edge weights correspond to the couplings and potentials in equation (1).

Like GGMs, their continuous counterpart, MGMs learn the conditional independence structure of a given set of features together with parameters that define the joint distribution of both continuous and discrete variables (Lee and Hastie 2015). The conditional independence structure is encoded in an undirected graph where nodes represent features and edges the conditional dependencies between them. The conditional distribution of a node (feature) *x_j_* given all other nodes (features) $x_{\setminus j}$ only depends on the values of the nodes that are directly connected to *x_j_*. More formally, the data are modeled as a pairwise Markov random field with density where $x_{1},\ldots ,x_{p}$ are continuous features and $y_{1},\ldots ,y_{q}$ discrete features where *y_j_* has *L_j_* distinct states. Together, the *x_j_* and *y_j_* form the nodes of the network. The remaining parameters are node and edge weights (couplings) that jointly define how the distribution of a node depends on the values of its direct neighbors. *β_js_* are couplings between two continuous nodes, *α_j_* are continuous node potentials, $\rho_{js}(y_{s})$ are continuous-discrete couplings, and $\phi_{js}(y_{j},y_{s})$ are discrete–discrete couplings. We denote the complete parameter set by $\Theta =\{\{\beta_{js}\},\{\alpha_{j}\},\{\rho_{jt}\},\{\phi_{rt}\},j,s\in \{1\ldots p\},r,t\in \{1\ldots q\}\}$. Figure 1 visualizes the roles of individual parameters.


(1) $$\begin{array}{c} p(x,y;\Theta )\propto \;\text{exp}\;(\sum_{j=1}^{p}\sum_{s=1}^{p}-\frac{1}{2}\beta_{js}x_{j}x_{s}+\sum_{j=1}^{p}\alpha_{j}x_{j}\\ +\sum_{j=1}^{p}\sum_{s=1}^{q}\rho_{js}(y_{s})x_{j}+\sum_{j=1}^{q}\sum_{s=1}^{q}\phi_{js}(y_{j},y_{s})), \end{array}$$

To simplify notations, we will omit the index *i* of the sample whenever the focus is on the features *x_j_* in the continuous and *y_j_* in the discrete case. Single data points in our data matrix are realizations of the random variables *x_j_* or *y_j_* and are denoted by *x_ij_* or *y_ij_*, respectively.

Equation (1) defines the full joint distribution of both discrete and continuous features. To judge whether a specific continuous *x_ij_* or discrete *y_ij_* data point fits to all other observed data points in the same sample, we need to calculate the conditional distribution of a node given all its direct neighbors. Following (Lee and Hastie 2015) the conditional distribution of a continuous variable *x_j_* given all other continuous variables $x_{\setminus j}$ and discrete variables *y* is Gaussian with where the linear regression yields the mean and the variance is given by $\beta_{jj}^{-1}$.


(2) $$\begin{array}{c} x_{j}|(x_{\setminus j},y;\Theta )∼N({\hat{x}}_{j},\beta_{jj}^{-1}) \end{array}$$

(3) $${\hat{x}}_{j}=\alpha_{j}+\sum_{s}\rho_{js}(y_{s})-\sum_{s\neq j}\beta_{js}x_{s}$$

The conditional distribution of a discrete variable *y_j_* with *L_j_* states has the probability mass function which corresponds to a multiclass logistic regression. Together, the conditional distributions (2) and (4) describe the conditional independence structure via the regression coefficient of a variable on all others. We denote the conditional distribution (2) of a continuous feature *x_j_* in a sample *i* by *Q_ij_* and the conditional distribution of a discrete feature *y_j_* in sample *i* by *p_ij_*.


(4) $$\begin{array}{c} p(y_{j}|y_{\setminus j},x;\Theta )=\\ \frac{\;\text{exp}\;\left(\sum_{s}\rho_{sj}(y_{j})x_{s}+\Phi_{jj}(y_{j},y_{j})+\sum_{s\neq j}\Phi_{js}(y_{j},y_{s})\right)}{\sum_{l=1}^{L_{j}}\;\text{exp}\;\left(\sum_{s}\rho_{sj}(l)x_{s}+\Phi_{jj}(l,l)+\sum_{s\neq j}\Phi_{js}(l,y_{s})\right)} \end{array}$$

### 2.2 Detection of data anomalies in continuous features

ADMIRE builds on the discrepancies between the original observations *x_ij_* from their model-based conditional distributions and the resulting linear predictions ${\hat{x}}_{ij}$. The estimated means ${\hat{x}}_{ij}$ from the conditional distribution (2) serve as a regression-based re-estimation of a continuous feature based on all other features (see Altenbuchinger *et al.* 2019). Furthermore, the conditional distribution describes how well an observed data point fits to the rest of the data. More specifically, it tells us the probability of observing a specific feature value given all other continuous and categorical features for the same sample. Let *x_ij_* be the observed, measured value, ${\hat{x}}_{ij}$ the estimated mean, and $\epsilon =|x_{ij}-{\hat{x}}_{ij}|$ the deviation of the observed value from the estimated mean. Then the probability *p* of observing a deviation greater or equal *ϵ* is given by where *F* is the cumulative distribution function of $x∼N({\hat{x}}_{ij},\beta_{jj}^{-1})$. We apply (5) to all entries *x_ij_* in the data matrix and rank them according to their probability. Entries at the top of this list have a low probability and are most likely anomalies. Mind that the same ranking is achieved, when instead of the probabilities the scores $s_{ij}^{o}=\left|x_{ij}-{\hat{x}}_{ij}\right|/\sqrt{\beta_{jj}^{-1}}$ are used for ranking. Data entries with a high deviation from the estimated mean rank at the top of the list.


(5) $$p=P(x\leq \hat{x}-\epsilon )+P(x\geq \hat{x}+\epsilon )=2*F(\hat{x}-\epsilon ),$$

We threshold this list by comparing the observed scores with anomaly-free scores simulated from the estimated distribution (2). For every observed data point *x_ij_*, let *Q_ij_* be its model-based conditional distribution given all other features k≠j of sample *i* defined in (2). We generate random data by drawing one random value *r_ij_* from each *Q_ij_*, resulting in as many random data points as original continuous observations. Note that this data does not contain anomalies, since every simulated data point was drawn from its proper conditional distribution. Let $s_{ij}^{r}=\left|r_{ij}-{\hat{x}}_{ij}\right|/\sqrt{\beta_{jj}^{-1}}$ be the score of *r_ij_*. The joint distribution of the $s_{ij}^{r}$ represents a score distribution for data in which no anomalies exist. Next, we sort the lists of observed scores $s_{ij}^{o}$ and random scores $s_{ij}^{r}$ and compare them rank by rank. If the real data contains anomalies, the scores of top ranking data points are higher than rank matching random scores. This results in different score distributions for highly ranking scores. To stabilize the distribution of random scores, we draw repeatedly from the distributions *Q_ij_* and compute $s_{ij}^{r}$ by averaging the resulting scores rank by rank. The first random score that exceeds its matched observed score is chosen for thresholding the lists and we flag all data points with an observed score higher than this threshold value as anomalies.

### 2.3 Detection of discrete anomalies

Similar to the continuous case, we can calculate for each discrete data entry *y_ij_* a score depending on the conditional distribution (4) and compare the resulting ranked list to anomaly-free scores generated from the estimated distribution.

Let $y_{ij}=k$ be the *j*th discrete feature in sample *i* with observed state *k*. Then the discrete observed score is defined as $s_{ij}^{o}=-\text{log}(p_{ij}(k))$, where $p_{ij}(k)$ is the conditional probability (4) of observing state *k* in feature *y_j_* for sample *i* given all other features (discrete and continuous). If the probability of observing $y_{ij}=k$ is low, the score $s_{ij}^{o}$ is high and the discrete feature is most likely erroneous. For thresholding, we draw for each observed discrete value *y_ij_* a random value *r_ij_* from the conditional distribution *p_ij_*. If the observation $y_{ij}=k$ is an anomaly, the probability $p_{ij}(k)$ of observing state *k* should be low, resulting in a realization $r_{ij}\neq k$ with a different state. We define random scores by $s_{ij}^{r}=-\text{log}(p_{ij}(r_{ij}))$. The random scores contain no anomalies. Again, we draw multiple times from the distribution and average over the repeated scores rank by rank. In line with the continuous case, we match observed and random scores rank by rank and set the threshold as the first random score that is higher as its observed counterpart.

### 2.4 Imputation of missing values

ADMIRE imputes missing values by a two-step procedure. If the value of feature *j* is missing in sample *i*, ADMIRE pre-imputes it in step 1 by the value of *j* in the sample i′, which has the smallest Euclidean distance to *i* among all samples where the value of *j* is not missing. After the pre-imputation, feature *j* is re-scaled in the entire dataset. In step 2, an MGM is fitted on the pre-imputed dataset including calibration of the regularization parameter. Finally, all pre-imputed missing values are re-estimated, as described in Sections 2.2 and 2.3.

### 2.5 Implementation and model training

ADMIRE estimates the parameter set $\Theta =\{\{\beta_{js}\},\{\alpha_{j}\},\{\rho_{jt}\},\{\phi_{rt}\},j,s\in \{1\ldots p\},r,t\in \{1\ldots q\}\}$ which defines the node and edge weights and hence specifies the joint probability distribution (1) together with the conditional distributions (2) and (4). Let ${\left\{x_{j}\right\}}_{j=1,\ldots p}$ be the standardized continuous features with mean 0 and variance 1 across samples and ${\left\{y_{j}\right\}}_{j=1,\ldots q}$ the discrete features. Then, following Altenbuchinger *et al.* (2019) and Lee and Hastie (2015), we minimize the negative pseudo log-likelihood to estimate Θ. The pseudo-likelihood (6) consists of the product of all conditional distributions where $Q(x_{s}|x_{\setminus s},y;\Theta )$ is the conditional distribution of a continuous variable given all other variables (2) and $p(y_{r}|x,y_{\setminus r};\Theta )$ is the distribution of a discrete variable conditioned on all other variables (4). The term $\lambda {\left\|\Theta \right\|}_{1}$ corresponds to the lasso penalty with an additional weighting scheme to adjust for group sizes and variances of the features (see Altenbuchinger *et al.* 2019). Following Altenbuchinger *et al.* (2019), the minimization is done using a proximal gradient descent algorithm (O’Donoghue and Candès 2015).


(6) $$\begin{array}{c} \tilde{l}(\Theta |x,y)=\\ -\sum_{j=1}^{p}\;\text{log}\;(Q(x_{j}|x_{\setminus j},y;\Theta ))-\sum_{j=1}^{q}\;\text{log}\;(p(y_{j}|x,y_{\setminus j};\Theta ))+\lambda {\left\|\Theta \right\|}_{1} \end{array}$$

The sparseness parameter *λ* is calibrated by leave-one-out cross-validation. More precisely, let $\lambda =\left(\lambda_{1},\ldots ,\lambda_{m}\right)$ be a sequence of values and i∈{1,…n}. For every *λ_k_* and every *i*, we fit a MGM leaving out the *i*th sample. The resulting parameters $\Theta_{i}(\lambda_{k})$ are used to re-estimate the continuous features *x_ij_* via Equation (3). For every *λ_k_*, we get a matrix ${\hat{x}}_{ij}$ with the same dimension as the continuous input data. We choose the *λ_k_* with smallest mean-squared error between original and re-estimated data as the optimal sparseness parameter. The corresponding parameters $\Theta_{i}(\lambda_{k})$ and the cross-validated estimators ${\hat{x}}_{ij}$ are finally used for anomaly detection.

Note that ${\hat{x}}_{ij}$ and ${\hat{y}}_{ij}$ are estimated given all other features in the sample and thus can be affected by other anomalies in the same sample. To compensate this effect, we check for each estimated data point ${\hat{x}}_{ij}$ in the continuous case or ${\hat{y}}_{ij}$ in the discrete case, if its regressors *x_ik_* and *y_ik_*, k≠j, are potential anomalies [probability (5) of <5%]. If a continuous estimator *x_ik_* is flagged as a potential anomaly, we replace it by the group mean ${\bar{x}}_{lk}$ where *l* corresponds to the samples with the same discrete states as sample *i*. If a discrete estimator *y_ik_* is flagged as an anomaly, we replace its state by the state with highest estimated probability. The resulting adjusted estimators then are used in (3) and (4) to predict ${\hat{x}}_{ij}$ and ${\hat{y}}_{ij}$.

ADMIRE is implemented in an easy-to-use Python package called adadmire which is listed in the python package index PyPi.

## 3 Simulations

We studied the performance of ADMIRE by simulating artificial anomalies in a proteomics dataset (Higuera *et al.* 2015). The dataset consists of protein expression levels from the brains of mice with and without Down syndrome. In total, 77 proteins (continuous features) were measured using reverse-phase protein arrays in several groups of mice that can be characterized by three discrete features: genotype (normal/trisomic), treatment (saline/memantine), and behavior: a protocol used to stimulate learning (shock-context/context-shock). In total, 72 mice were analyzed with three replicates in a five-point dilution series resulting in 1080 measurements per protein. Each measurement can be considered as an independent sample. Since the focus of this study is the evaluation of ADMIRE’s anomaly detection and correction, we excluded 12 proteins because they contained missing values. Extensive performance evaluation of ADMIRE’s imputation routine can be found in Supplementary data. Furthermore, we sub-sampled 400 samples such that each of the eight different groups of mice was represented by 50 samples. This resulted in a dataset of 400 samples, 68 continuous features, 3 discrete features, and 400 * 68 = 27 200 continuous and 3 * 400 = 1200 discrete data points. In the following analyses, we used the log-transformed protein measurements. Further information on the dataset can be found in Supplementary data.

### 3.1 Anomaly detection

To validate the detection of discrete anomalies, we introduced artificial anomalies by changing the original states of the discrete features. For each feature we chose two samples and swapped the according states, e.g. a sample with original treatment “Saline” was assigned to the other treatment state “Memantine.” Thereby, we introduced six artificial anomalies in the dataset.

**Figure 2.**
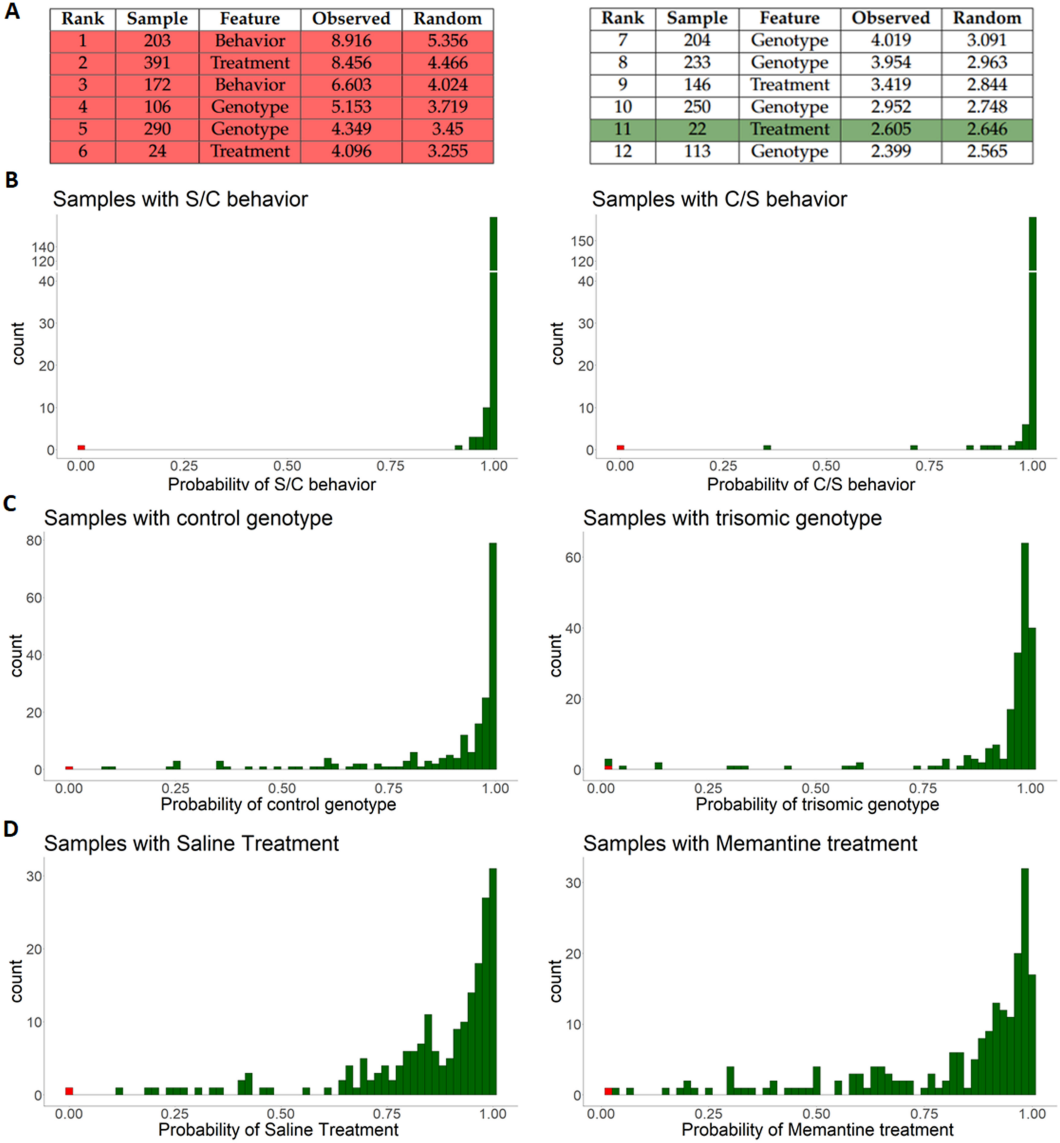
Observed and random scores for the dataset containing artificial discrete anomalies and estimated probabilities for the categorical variables split in the respective binary states across the according samples. (A) Highest ranking observed and random scores, artificial anomalies are marked in red, the threshold is marked in green. (B) Estimated probabilities for behavior (C/S or S/C). (C) Estimated probabilities for genotype (control/trisomic). (D) Estimated probabilities for treatment with treatment either Memantine or Saline.

ADMIRE detects among the 1200 discrete data points 10 anomalies. Figure 2A reports the 12 discrete data points with highest ranking observed scores. Additionally, we reported for each rank the corresponding calculated random score. In green, we marked the threshold for anomaly detection, where the random score exceeds the equally ranking observed score. The rows marked in red correspond to the artificially introduced anomalies. As can be seen, all six artificially introduced anomalies are detected by ADMIRE. The other detected anomalies cannot be verified since the dataset was not generated by us. Figure 2B–D additionally shows the estimated probabilities for the three features split in their corresponding states. Overall, high probabilities (low scores) were computed for all data points, except for the samples where the state was swapped (marked in red).

**Figure 3.**
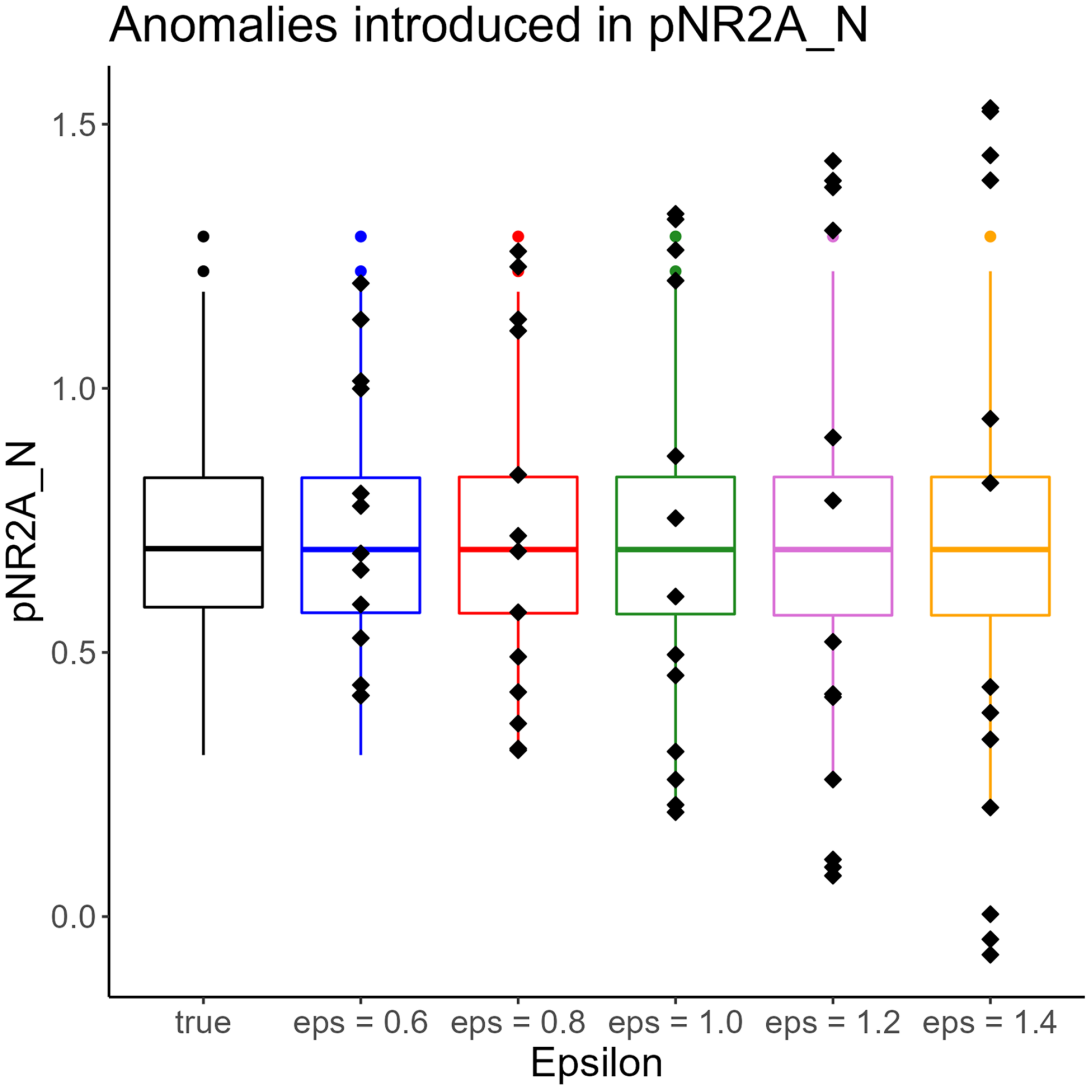
Influence of the parameter *ϵ* on the strength of the anomalies in protein pNR2A_N. Black dots indicate introduced anomalies.

To study anomaly detection in continuous data points, we introduced artificial anomalies similar as in Steinbuss and Böhm (2017). We randomly choose *n_a_* data points and perturb them by adding random shifts. The size of the shifts is relative to the normal range of the feature and can be calibrated by a parameter *ϵ*. For ϵ<1, the perturbed data does not exceed the range of the feature and thus does not present an outlier. For larger values of *ϵ*, the perturbations can introduce outlier values as well. In addition, our simulation ensures that every chosen data point is perturbed by at least 15%. Details on the simulation can be found in Supplementary data. For illustration, Fig. 3 shows the distribution of artificial anomalies introduced in the data of the protein pNR2A_N for different values of *ϵ*. We ran 10 simulation scenarios varying the number of introduced anomalies and their strengths *ϵ*. We either introduced 2.5% anomalies (corresponding to 680 perturbed data points) or 5% (corresponding to 1360 perturbed data points) and also varied the strength *ϵ* of the introduced anomalies. In Supplementary Table 2, we summarized the 10 simulations.

**Figure 4.**
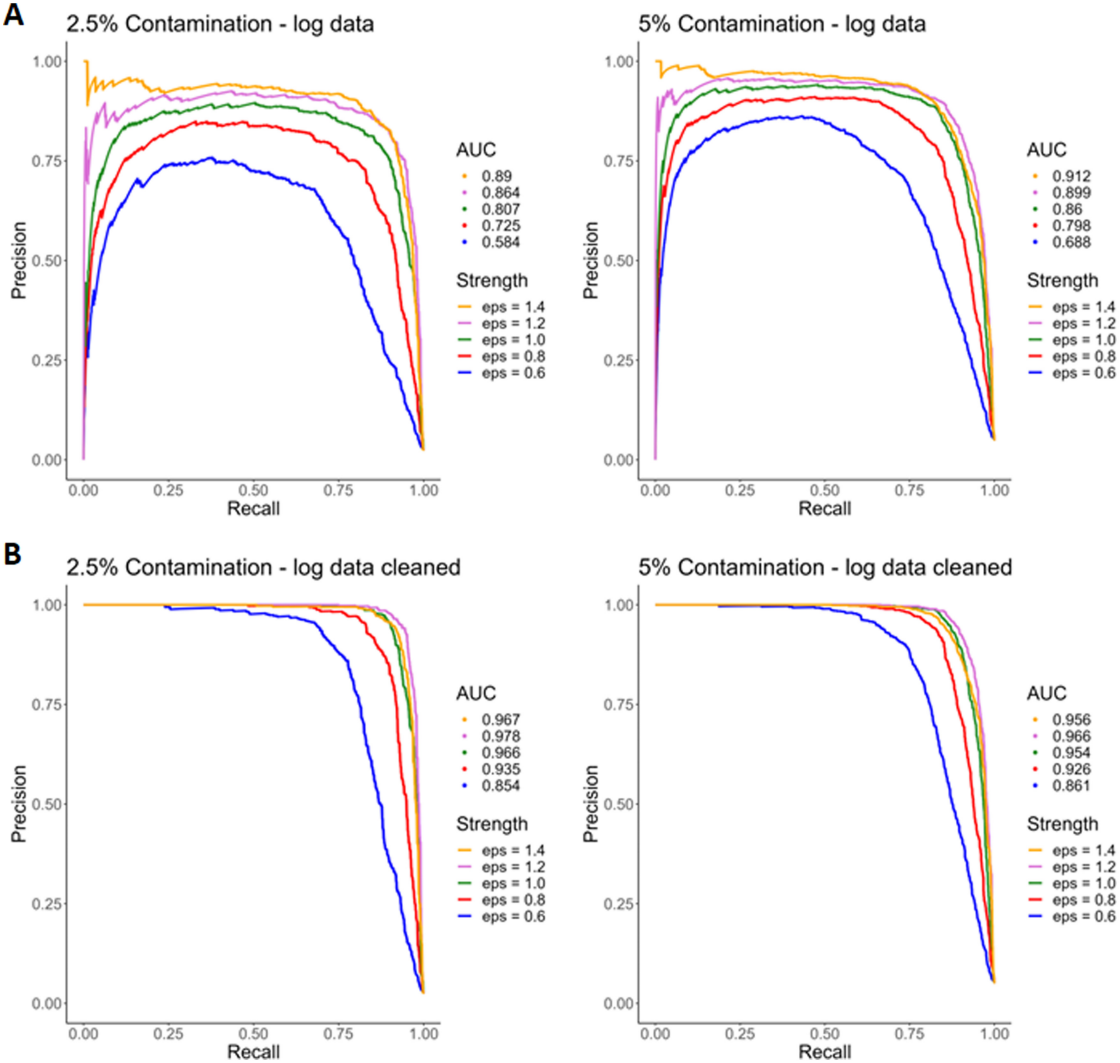
Precision–Recall curves for the simulations with 2.5% and 5% contamination. (A) PR curves of ADMIRE on log-transformed data without correcting for intrinsic outliers. (B) PR curves of ADMIRE on log-transformed simulations corrected for intrinsic outliers.

The algorithm shows good performance in the detection of anomalies with an area under the curve of 0.890 for a contamination level of 2.5% and of 0.912 for 5% contamination and *ϵ* set in both cases to 1.4. With decreasing *ϵ* (1.2 − 0.6), the magnitude of the anomalies decreases and the number of hidden anomalies increases. Therefore, the anomalies are harder to detect, which is reflected in lower AUCs. Nevertheless the detection of anomalies remains good with AUCs ranging from 0.864 to 0.584 for 2.5% contamination and 0.899 to 0.688 for 5% of contamination (see Fig. 4A). Note, that we did not adjust the proteomics data for intrinsic anomalies that might exist in addition to the simulated ones. If we did identify these anomalies using ADMIRE and adjust the PR curves for them (see Fig. 4B), the performance increases further, with AUCs now ranging from 0.978 to 0.854 for 2.5% of contamination and 0.966 to 0.861 for 5% contamination. Further information on the detection of intrinsic anomalies can be found in Supplementary data.

Finally, we compared ADMIRE to three competing outlier detection algorithms: Isolation Forest (Liu *et al.* 2008), LOF (Breunig *et al.* 2000), and stray (Talagala *et al.* 2021) in the context of the 10 simulations described above. Since these methods aim at finding anomalous instances in a dataset, we applied them feature-wise. Our algorithm outperforms all methods, which reached only maximal AUCs of 0.63 and 0.747 for 2.5% and 5% contamination (stray) and 0.701 and 0.789 (LOF) on the log-transformed simulations. Isolation Forest performed best on the scaled raw data with AUCs up to 0.828 for 2.5% and 0.888 for 5% contamination. Further information on how Isolation Forest, LOF, and stray were applied can be found in Supplementary data, together with the precision recall curves after correcting for the intrinsic anomalies.

### 3.2 Anomaly correction

Here we study how ADMIRE performs in correcting detected anomalies. For the 10 simulations described above, we calculated anomaly thresholds and corrected all data points by replacing them with their re-estimated values (3). We next compared both the uncorrected (perturbed) and corrected data to the original data (ground truth) and calculate mean absolute percentage errors for both (Table 1). Anomaly correction reduced theses errors strongly, showing that the algorithm automatically can improve the quality of datasets significantly. Note that correction was applied to all detected anomalies including the falsely detected ones, suggesting that in case of false-positive detections, the corrections do not compromise the data very much.

**Table 1.** Summary of the corrected datasets.^a^

Dataset	*ϵ*	# Introduced	# Detected	TP	$MAPE_{i}$ (%)	$MAPE_{c}$ (%)
*S*_1_	0.6	680	856	504	1.047	0.823
*S*_2_	0.6	1360	1244	927	2.073	1.22
*S*_3_	0.8	680	812	566	1.396	0.747
*S*_4_	0.8	1360	1244	1040	2.763	1.139
*S*_5_	1.0	680	763	596	1.746	0.676
*S*_6_	1.0	1360	1241	1103	3.454	1.119
*S*_7_	1.2	680	729	606	2.095	0.65
*S*_8_	1.2	1360	1198	1093	4.145	1.23
*S*_9_	1.4	680	666	578	2.444	0.73
*S*_10_	1.4	1360	1072	1008	4.836	1.473

a The table shows the strength of the simulation (*ϵ*), the number of introduced anomalies (column “# Introduced”), the number of detected continuous anomalies (column “# Detected”) and the number of true-positive anomalies among the detected ones (TP), the anomaly simulation introduced mean average percentage error (${\text{MAPE}}_{i}$) and the mean average percentage error after correcting the datasets with ADMIRE (${\text{MAPE}}_{c}$).

## 4 Anomaly detection in metabolomics data

We used ADMIRE to investigate anomalies in one of our own metabolomics datasets (Feist *et al.* 2018). These data were generated to study the metabolism of B-cells in response to stimuli from a tumor micro-environment. In particular, we were interested how the responses changed when the oncogene MYC was activated. MYC activation is a hallmark of many B-cell lymphomas. We used human P493/6 B-cells that contain an inducible MYC-construct and stimulated them with different cocktails of micro-environmental factors. Their metabolism responded to these stimuli and we profiled these changes using both nuclear magnetic resonance (NMR) spectroscopy and mass spectrometry (MS) applied to the cell cultures’ supernatants and cell pellets, which were both independently measured. Note that in the previous paper by Feist *et al.* (2018), only cell pellet data were evaluated, while the present contribution focuses on the data obtained from the corresponding supernatants.

Continuous features consist of 49 metabolites that were quantified in a total of 100 samples. 11 features were measured using NMR and 38 using MS. The discrete features are the MYC status (high/low) of the B-cells and the 10 batches in which the samples were processed.

We ran ADMIRE on the full dataset including both continuous and discrete variables. First, we checked for discrete anomalies. These could be manual data entry mistakes such as misassignments of either the MYC-status or one of the batches. Supplementary Fig. 8A shows observed scores next to rank matching random scores for the 10 top scoring discrete data points. No observed score exceeds the random score and we conclude that all discrete features are correct. Artificially introduced errors, similar to Section 3, were detected correctly, see Supplementary data.

Next, we studied potential anomalies in the continuous metabolite measurements. Our algorithm flagged 46 out of 4900 continuous data points as anomalies (0.94%). The flagged anomalies are distributed uniformly across the 49 features with mostly only one anomaly per feature, indicating that there are no globally conspicuous features. However, if we mapped anomalies to samples, a different distribution was observed. Supplementary Fig. 9 shows that while most samples contain only a small number of anomalies (75% of the sample do not even have an anomaly at all), two samples show significantly more. In sample 7, ADMIRE flagged 11 out of the 49 continuous features as anomalies and in sample 92, a total number of 7 features were flagged.

**Figure 5.**
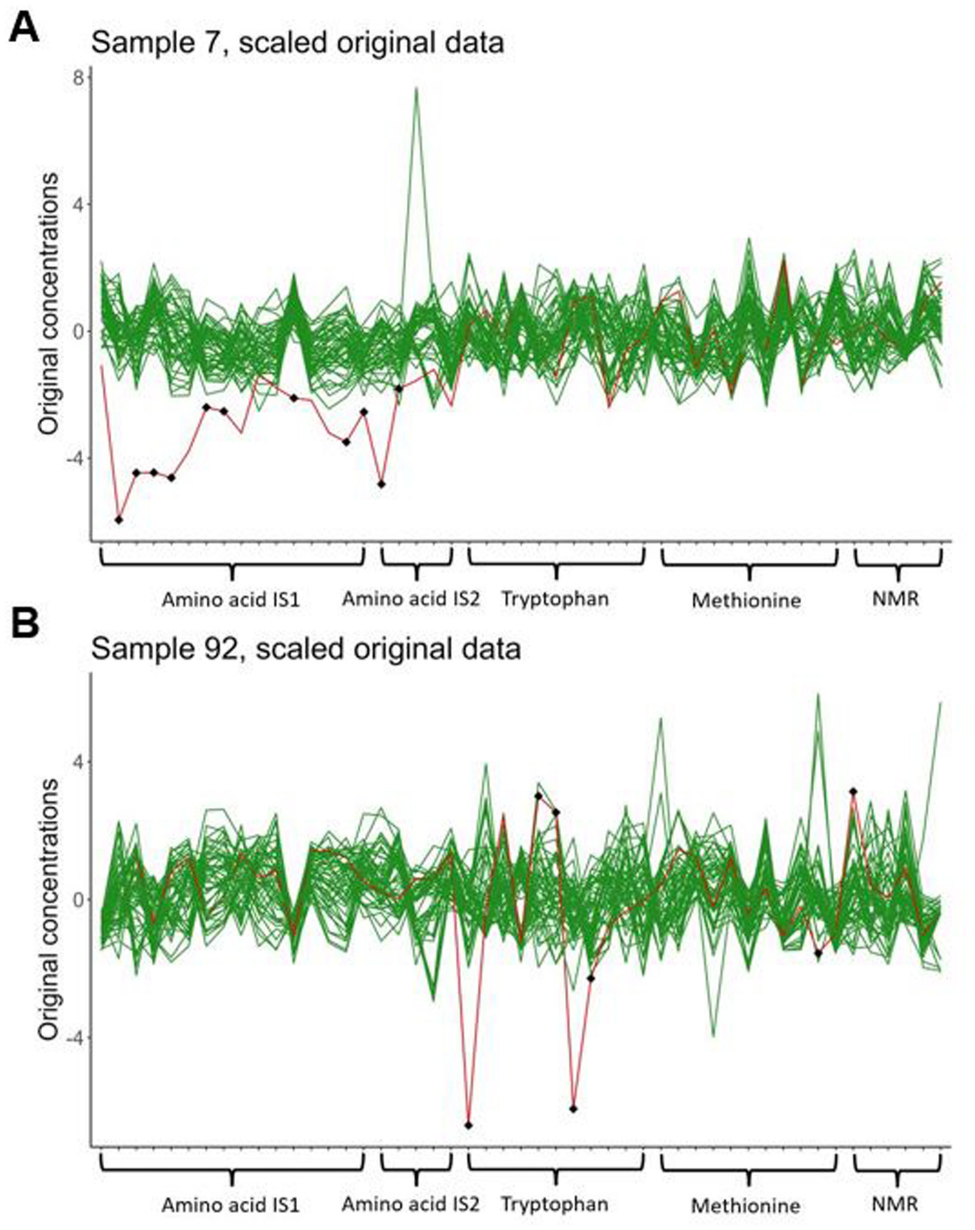
(A) Scaled, originally measured concentrations of sample 7 (red) with all other samples in the same MYC group (green), detected anomalies are marked as black diamonds. The features (metabolites) on the *x*-axis are ordered according to the different quantification methods. (B) Scaled, originally measured concentrations of sample 92 (red) with all other samples in the same MYC group (green), detected anomalies are marked as black diamonds. The features (metabolites) on the *x*-axis are ordered according to the different quantification methods.

Figure 5A shows sample 7 (red) together with all samples of the same MYC state (green lines). The black diamonds are the anomalies detected by ADMIRE. All anomalies are in the first two blocks, which correspond to the metabolites that where quantified by MS. All of them were amino acids. To verify that the detected anomalies are genuine errors, we quantified them again using NMR, a completely independent method. This was possible for 10 out of 11 flagged features. Only for cystine NMR signals were too low and highly overlapping such that no NMR measurement was possible. For the remaining 10 metabolites, NMR confirmed that the MS-based measurements were in fact incorrect, deviating by more than 15% from the corresponding NMR measurements. We suspect that a pipetting mistake in the probe preparation for amino acid MS is responsible for the anomalies ADMIRE found. Metabolites were quantified relative to added internal standards with different separate standard mixes for amino acids and tryptophan and therefore, any pipetting error in the standard will falsify results for this specific measurement type. Further, note that for each measurement method such as the amino acid method or the tryptophan method, a separate internal standard mix was used. As a consequence, a pipetting error can be detected using NMR as a validation method since it uses a different internal standard and is, therefore, not affected. This shows nicely the potential of the MGM for detecting true anomalies and also patterns of anomalies within a sample.

For the validation of the anomaly correction, we calculated the mean absolute percentage error for the 11 anomalies of sample 7 with clear NMR signals. Hereby we used for cystine, that could not be validated by NMR, the originally measured concentration. The MAPE between the originally measured and validated values is reduced from 76.63 to 12.27 when the originally measured values were replaced by the corrections proposed by ADMIRE.

The sample with the second highest amount of anomalies is sample 92. In this sample ADMIRE detected seven anomalies. Only two of these could be quantified by NMR (one false positive and one true anomaly). For the other metabolites, NMR signals were too low and overlapping for accurate quantification. Figure 5B shows sample 92 together with all other samples of the same MYC state. The anomalies are mostly located in the tryptophan group of measurements, which was independently measured employing a dedicated MS method (see Supplementary data for details). Again, this points to a possible pipetting error during sample preparation. Most probably, the sample volume used for the tryptophan method was incorrect.

For the remaining flagged anomalies, we inspected the raw spectra and searched for deviations or errors in the integration of the single spectra. Whenever possible, we validated MS measurements by re-analyzing the correspondent NMR spectra. This is only possible for metabolites with concentrations up to a lower limit of micromolecular range. For smaller concentrations, the sensitivity of the NMR is not sufficient enough to quantify reliably. Table 2 reports all 46 anomalies sorted by their anomaly score. The last three columns show the corrections proposed by ADMIRE, the originally measured value (original) and the validated, true measurement (validated), respectively. All anomalies that could be unambiguously validated as anomalies are highlighted in green. For them, the difference between the original and the verified value was at least 15%. False positives, where ADMIRE detected an anomaly but the verification showed no erroneous measurement or other peculiarity are marked in red. Note that we treated metabolites that couldn’t be verified by an independent method and whose spectra showed no abnormalities also as false positives. These anomalies are marked with an asterisk. The rows highlighted in yellow correspond to the anomalies of sample 92 which all belong to the tryptophan measurement group. Here, we couldn’t verify an error in the measurement, but a mishap during sample generation similar to sample 7 is likely. Two anomalies belonging to the features Spermidine and 3-Hydroxyanthranilic acid are marked in purple. We included these two features although both contained a large number of imputed values and measurements below the lower limit of quantification. Note that these values were not imputed by ADMIRE but preprocessed using the laboratory’s own pipeline.

**Table 2.** Detected anomalies ordered according to their scores.^a^

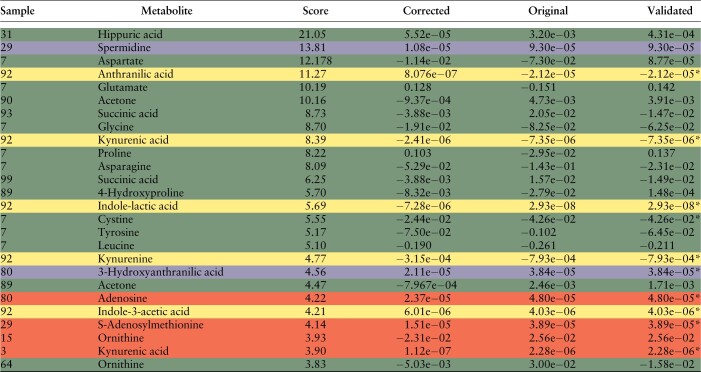
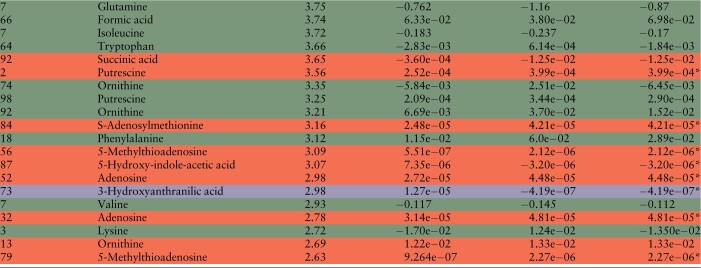

a Rows marked in green are anomalies that could be validated as true anomalies. Red corresponds to measurements that either show no conspicuous MS spectra but could not be validated by an independent method (marked with *) or false positives where the original measurement is correct. Yellow corresponds to the anomalies in sample 92 that could not be validated by an independent method and purple to anomalies where the original data contained a large amount of imputed values. All concentration values are given in mM.

We calculated for the 46 validated data points in Table 2 the MAPE between the original measured concentrations and the validated concentrations and compared it to the MAPE between the corrections proposed by ADMIRE and the validated ones. Using the corrected concentrations, the MAPE decreased from 23.015 to 10.802, which is an almost 2.5-fold improvement. Again, the false-positive anomalies were included in the calculation of the MAPE. This shows once more that even if ADMIRE detects a false-positive anomaly, its correction is still close to the original, true value.

## 5 Discussion

Incorrect data points make data analysis invalid, even if they are infrequent. In large datasets, they are hard to detect manually, but easier to detect automatically because they are inconsistent with the inherent structure of the rest of the data. Here we describe ADMIRE, an algorithm that combines MGMs and cross-validated re-estimation of data points to detect data anomalies in large mixed molecular datasets. The MGM learns inherent data structure, the CV-based re-estimation checks whether individual data points are consistent with this data structure.

Outliers are a special instance of anomalies. An outlier is a value of a feature that is suspiciously higher or lower than all other values of the same feature. In general, they are more easily detected. Although we can in principal detect them feature by feature independently from all other features, the use of conditional distributions can nevertheless support the process. Importantly, anomalies do not need to present as univariate outliers and in fact many of the anomalies we detected did not.

ADMIRE was primarily designed for molecular datasets that combine continuous features such as abundance of certain molecules (OMICS data) with discrete features that for example describe experimental designs or patient characteristics. Here, incorrect data in continuous features can result from experimental artifacts, while incorrect discrete data can be caused by incorrect manual data entry. However, ADMIRE can be used for any large dataset continuous, discrete, or mixed.

ADMIRE does not only detect anomalies, but it also has routines to correct them thus generating more consistent datasets. In this way, it can be used as a pre-processing or data normalization routine as well. Additionally, the adadmire package offers a testing routine that allows the user to test ADMIRE in simulations with their own data. Finally, anomalies do not need to be incorrect data points. They can also be observations that are rare, unusual but correct. Such oddities can be scientifically interesting and ADMIRE can be used to spot them for further investigation. In this way, it can be used as a data mining tool as well.

## Supplementary Material

btad501_Supplementary_Data
