# Supplementary material for: Anomaly detection in mixed high-dimensional molecular data
Source: Bioinformatics. 2023 Aug 16;39(8):btad501. doi: 10.1093/bioinformatics/btad501 (PMC10457663; doi:10.1093/bioinformatics/btad501)
Supplement: btad501_Supplementary_Data [file btad501_supplementary_data.pdf]

Anomaly detection in mixed high dimensional molecular data - Supplement

Lena Buck[1,*], Tobias Schmidt[1], Maren Feist[2], Philipp Schwarzfischer[3], Dieter Kube[2], Peter J. Oefner[3], Helena U. Zacharias[4], Michael Altenbuchinger[5], Katja Dettmer[3], Wolfram Gronwald[3,*], and Rainer Spang[1,*]
[1]Department of Statistical Bioinformatics, University of Regensburg, 93040, Regensburg, Germany.
[2] Department of Hematology and Medical Oncology, University Medicine Goettingen, 37075 Goettingen, Germany.
[3]Institute of Functional Genomics, University of Regensburg, 93040, Regensburg, Germany.
[4]Peter L. Reichertz Institute for Medical Informatics of TU Braunschweig and Hannover Medical School, Hannover Medical School, 30625 Hannover, Germany.
[5]Department of Medical Bioinformatics, University Medical Center Göttingen, 37075 Göttingen, Germany.

Contents

1 Data and code availability **2**

2 Imputation **2**
2.1 Performance of imputation 2
2.2 Influence of missing values on anomaly detection 3
2.3 Features with high amount of missing values 4

3 Supplementary Information for the validation of ADMIRE in simulations **8**
3.1 Generation of artificial anomalies 8
3.2 Handling of intrinsic anomalies 9
3.3 Comparison to other anomaly detection methods 10

4 Supplementary information for the validation of ADMIRE on the metabolic data set **15**
4.1 Experimental setup 15
4.2 Detection of artificial discrete artefacts 20

4.3 Distribution of detected continuous anomalies in samples . . . 21

5 Performance dependency on contamination rate **21**

6 Data distribution **23**

7 Runtime analysis **25**

8 Visualization of Mixed Graphical Models **27**

1 Data and code availability

All data and code used in the manuscript can be found at https://github.com/spang-lab/adadmire. ADMIRE is implemented in a python package called adadmire which can be downloaded at https://pypi.org/project/adadmire.

2 Imputation

ADMIRE imputes missing values in a data set by applying a two-step procedure. First, it pre-imputes missing values by calculating for each sample the first nearest neighbour. Hereby, the pairwise euclidean distance is used where values that are missing in either sample are ignored and the weight of the remaining features is scaled up. If a sample contains missing values in some of its features they are pre-imputed with the corresponding values of its nearest neighbor. If some of these values are missing as well, the second nearest neighbor sample is used for them instead et cetera pp. This pre-imputation step works for continuous and categorical variables analogously and results in a data matrix containing no missing values.
Next, an MGM is fitted on the pre-imputed data set. The penalty parameter λ is calibrated by choosing the amount of regularisation corresponding to the minimal MSE between estimated and observed data values. Next, the pre-imputed missing values are re-estimated using equation (3) and (4) of the main text for continuous and discrete variables respectively together with the parameters obtained by the fitted MGM. These estimates then are used for the final imputation.

2.1 Performance of imputation

We compared the performance of imputation by the ADMIRE and the MissForest algorithms ([1]). MissForest is a non-parametric method that like ADMIRE can impute continuous and categorical features. We performed several simulations where we artificially introduced missing values in the data set from ([2]) which was pre-processed as described in the main text. The number of randomly introduced missing values ranged from 5% to 50% with continuous

and categorical features equally affected. Supplemental Table 1 shows the mean average percentage error (MAPE) between the original, true values and the imputed continuous features. For the categorical features we report the number of incorrectly imputed states. For continuous features ADMIRE outperformed MissForest in the range of 5% to 35% missig values, while for larger percentages MissForest performed better. For the imputation of discrete features ADMIRE outperformed MissForest in all cases.

**Simultaion**	**ADMIRE**	**MissForest**
5% (1360 cont., 60 discrete NAs)	MAPE continuous: 5.92, wrongly imputed states: 2	MAPE continuous: 6.78, wrongly imputed states: 3
10% (2720 cont., 120 discrete NAs)	MAPE continuous: 6.06, wrongly imputed states: 3	MAPE continuous: 6.80, wrongly imputed states: 5
15% (4080 cont., 180 discrete NAs)	MAPE continuous: 6.49, wrongly imputed states: 6	MAPE continuous: 7.09, wrongly imputed states: 11
20% (5440 cont., 240 discrete NAs)	MAPE continuous: 6.64, wrongly imputed states: 10	MAPE continuous: 7.29, wrongly imputed states: 13
25% (6800 cont., 300 discrete NAs)	MAPE continuous: 6.85, wrongly imputed states: 10	MAPE continuous: 7.32, wrongly imputed states: 17
30% (8160 cont., 360 discrete NAs)	MAPE continuous: 7.14, wrongly imputed states: 17	MAPE continuous: 7.48, wrongly imputed states: 29
35% (9520 cont., 420 discrete NAs)	MAPE continuous: 7.46, wrongly imputed states: 30	MAPE continuous: 7.71, wrongly imputed states: 34
40% (10880 cont., 480 discrete NAs)	MAPE continuous: 8.17, wrongly imputed states: 44	MAPE continuous: 8.06, wrongly imputed states: 50
45% (12240 cont., 540 discrete NAs)	MAPE continuous: 8.42, wrongly imputed states: 48	MAPE continuous: 8.31, wrongly imputed states: 57
50% (13600 cont., 600 discrete NAs)	MAPE continuous: 8.92, wrongly imputed states: 71	MAPE continuous: 8.52, wrongly imputed states: 72

Table 1: Imputation performance of ADMIRE and MissForest in simulations with 5% to 50% artificially introduced missing values (NAs).

2.2 Influence of missing values on anomaly detection

We investigated the impact of missing values on ADMIRE's ability to detect anomalies. We performed simulations with 5% and $\epsilon = 0.6, 1.0, 1.4$, (see simulations S_2, S_6 and S_{10}, respectively) and missing values ranging from 5% to 50%. The position of the missing values in the data set was randomly sampled resulting in an equally distributed spread across the data set. Note that the missing values were introduced such that they did not overlap with the artificially introduced anomalies. Next, we applied ADMIRE's imputation function to get estimates of the missing values. Afterwards, the anomaly detection routine of ADMIRE was used to detect the anomalies in the imputed data set.

The precision-recall (PR) curves and corresponding AUCs are reported in Supplemental Figure 1 A), C) and E). ADMIRE shows good performance in the detection of anomalies even if the data set includes a high amount of missing values. The AUCs range from 0.570 to 0.698 for the anomaly simulation with $\epsilon = 0.6$, from 0.865 to 0.778 for $\epsilon = 1.0$ and 0.916 to 0.826 for $\epsilon = 1.4$. Remarkable is that the AUC first increases when missing values were imputed (for example from 0.912 (0% missing values, baseline) to 0.916 (5% NAs) for $\epsilon = 1.4$). This can be explained by the fact that some intrinsic anomalies of the data set were set to missing values and afterwards imputed and thereby corrected by ADMIRE, resulting in less false positive anomalies and a higher AUC. To support this, we report the precision-recall curves and AUCs where the intrinsic anomalies found by ADMIRE in Supplemental Figure 1B), D), F) were excluded. As can be seen with increasing amount of introduced anomalies the AUCs monotonously decrease showing the true influence of the imputation on the anomaly detection. For 50% of missing values the lowest AUCs amounted for ϵ values of 0.6, 1.0, and 1.4 to 0.675, 0.847, and 0.860, respectively. This shows that ADMIRE can successfully cope with a large amount of missing values in a data set as long as the missing values are distributed equally across the data set.

2.3 Features with high amount of missing values

We evaluated the performance of ADMIRE in simulations where many missing values were introduced to the same feature. To this end, we randomly selected three proteins (pCREB_N, GSK3B_N, IL1B_N) from simulation S_6 (5% contamination and $\epsilon = 1.0$, see main text) and introduced between 10% and 90% missing values only in these three features, while ensuring that artificial anomalies were not replaced by missing values. Next, missing values were imputed using ADMIRE's imputation routine. The resulting data sets were used to assess the influence of missing values in a single feature on ADMIRE's anomaly detection performance. Supplemental Figure 2 A), B) and C) show results for the three proteins respectively. The constant black dashed line corresponds to the number of introduced artificial anomalies (22 artificial anomalies in pCREB_N, 23 in GSK3B_N and 18 in IL1B_N). The red line refers to the number of anomalies ADMIRE detected and the green line to the number of true positives. In this simulation we observed that for up to 60 % missing values, anomaly detection was hardly affected. If the number of imputed values was increased further, ADMIRE overestimated the number of anomalies. Concurrently, the number of true positives went down resulting in lower precision. Above 80% missing values ADMIRE broke down. We repeated the simulations with only half of the samples (200), see Supplemental Figure 3. We observed similar performance characteristics as with 400 samples, but now ADMIRE broke down with 50% missing values. Note that of the 200 samples 100 to 120 contained imputed values and of the remaining 100 to 80 samples up to 12 contained artificial anomalies and some additional intrinsic ones as well.

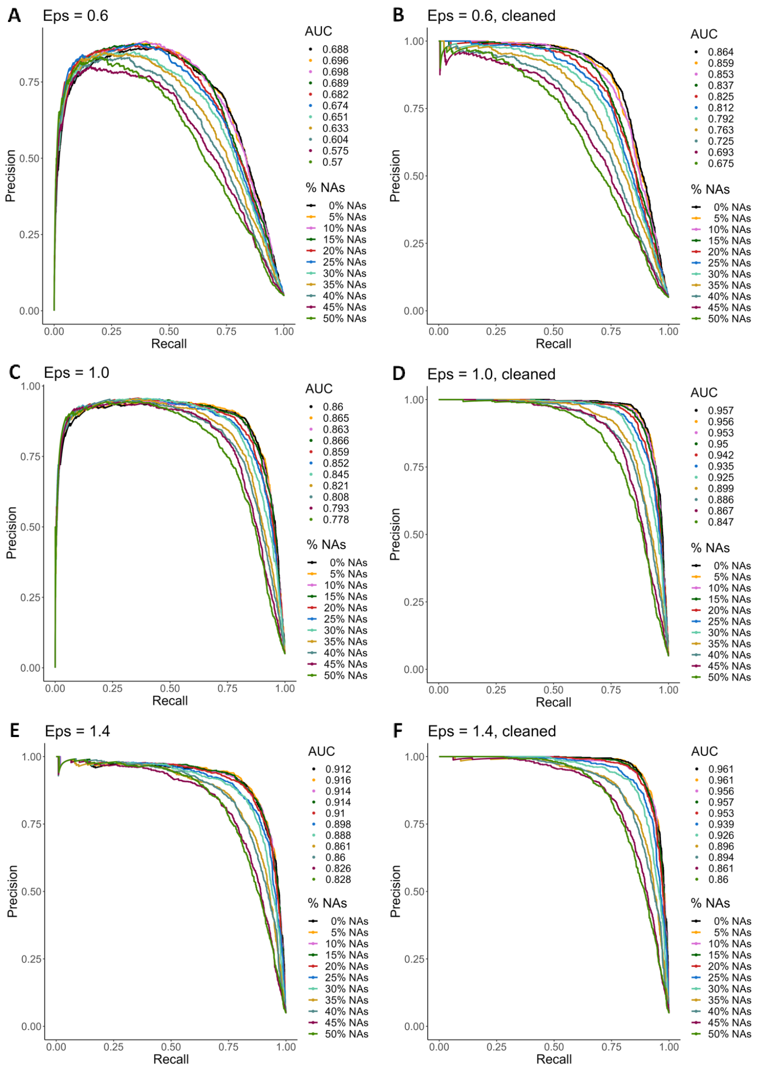

Figure 1: A), C), E) PR-curves and corresponding AUCs of the anomaly detection in a data set containing both missing values (from 0% to 50%) and artificially introduced anomalies (5% and ϵ = 0.6 (A), ϵ = 1.0 (C), ϵ = 1.4 (E), see main text). Missing values were first imputed using ADMIREs imputation routine, afterwards anomalies were detected by ADMIRE.
B), D), F) PR-curves and corresponding AUCs of the anomaly detection in a data set containing both missing values (from 0% to 50%) and artificially introduced anomalies (5% and ϵ = 0.6 (B), ϵ = 1.0 (D), ϵ = 1.4 (F) see main text) after correcting for intrinsic anomalies. Missing values were first imputed using ADMIREs imputation routine, afterwards anomalies were detected by ADMIRE.

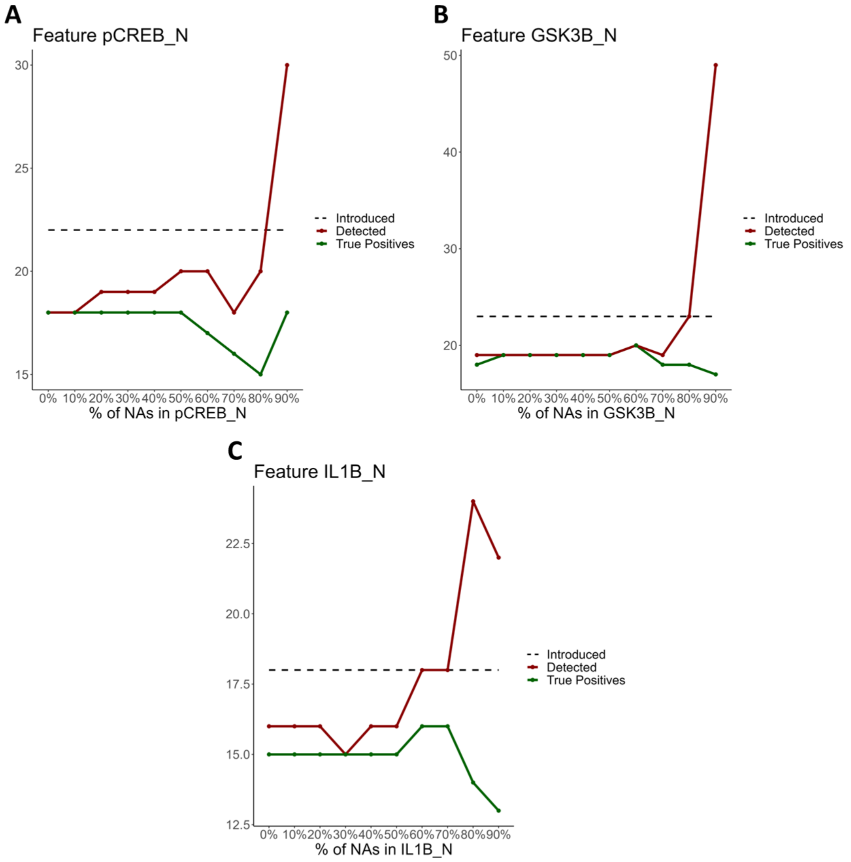

Figure 2: Simulations where between 10 and 90% of missing values were introduced in the values of the proteins pCREB_N (A), GSK3B_N (B) and IL1B_N (C) of the anomaly simulation data set S_{10} with $\epsilon = 1.0$ and 5% of artificially introduced anomalies. The black dashed lines indicate the number of artificially introduced anomalies in the corresponding feature, the red lines correspond to the number of anomalies detected by ADMIRE across the different simulations and the green lines to the true positives, the correctly detected anomalies.

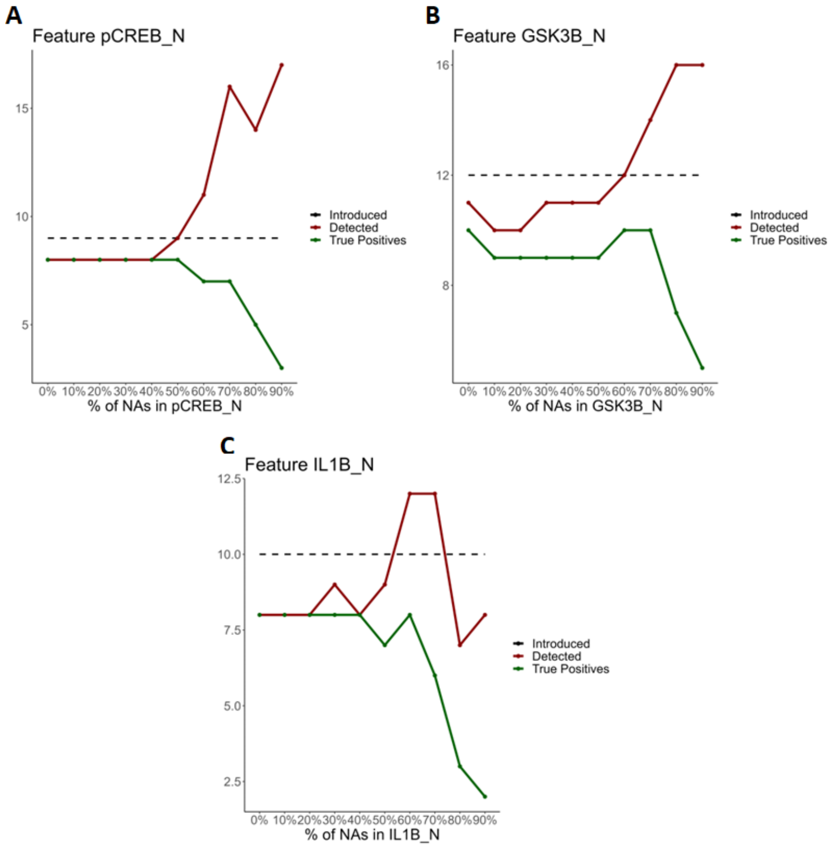

Figure 3: Same simulations as before but this time with 200 instead of 400 samples.

3 Supplementary Information for the validation of ADMIRE in simulations

3.1 Generation of artificial anomalies

Algorithm 1 Placing anomalies

Input: data set $X \in \mathbb{R}^{n \times p}, n_a, \epsilon \geq 0$
$X^s \leftarrow min\_max(X)$ ▹ feature-wise min-max transformation
Sample n_a random data points $x^s_{ij}, i \in \{1, \ldots, n\}, j \in \{1, \ldots, p\}$
for each of the sampled data points **do**
  $z \leftarrow \frac{0.15 x_{ij}}{max(x_{.j}) - min(x_{.j})}$
  $lowerChange \leftarrow$ sample from $Unif(z, x^s_{ij} + z)$
  $upperChange \leftarrow$ sample from $Unif(z, 1 - (x^s_{ij} + z))$
  Select at random if
  $\hat{x}^s_{ij} \leftarrow x^j_i - \epsilon \cdot lowerChange$
  $\hat{x}^s_{ij} \leftarrow x^j_i + \epsilon \cdot upperChange$
end for
scale anomalies $\hat{x}^s_{ij}$ back
return n_a Anomalies $\hat{x}_{ij}$

To validate the detection of anomalies in the continuous measurements we use the down-sampled proteomic data set described in the main text as ground truth and introduce artificial anomalies. For the anomaly generation we adapt the approach suggested by [3]. The authors propose an algorithm for outlier generation in high-dimensional spaces. Thereby, they focus on the introduction of so-called hidden outliers, which are only detectable in certain subspaces and located in regions close to existing data objects. The algorithm consists of two steps. In the first step, it shifts random data points in the data space and in the second step it filters for the hidden outliers. Since we are interested in hidden outliers as well as data points which are too high or low, the second step was neglected to introduce both types of anomalies. Our adapted version is summarized in algorithm 1. It requires three parameters: the feature-wise lower and upper bounds l, u of the data set such that it holds $X = (x_1, x_2, \ldots, x_p) \in [l, u]$, the number of anomalies n_a, which should be generated and the "strength" of the anomalies ϵ. Hereby, $X \in \mathbb{R}^{n \times p}$ denotes the data set, where n is the number of samples and p is the number of features per sample.
We first transform the data feature-wise to the interval $[0, 1]^p$ using the min-max standardization $x^s_{ij} = \frac{x_{ij} - min(x_j)}{max(x_j) - min(x_j)}$, therefore $l = 0$ and $u = 1$. Furthermore, to ensure that the generated anomalies deviate at least 15% from the original value, the interval from which the upper and lower changes are drawn is shifted such that the deviation is at least 15%. In case of $\epsilon < 1$ this cannot be guaranteed and we have to additionally filter out data points with a deviation of less than

15%. In case the data set contains only positive values there is also the option to select only anomalies that still reside in the positive range.

Simulation	Anomalies	ϵ	% hidden
S_1	2.5%	0.6	14.41%
S_2	5%	0.6	15.37%
S_3	2.5%	0.8	7.79%
S_4	5%	0.8	8.38%
S_5	2.5%	1.0	4.49%
S_6	5%	1.0	5.37%
S_7	2.5%	1.2	2.5%
S_8	5%	1.2	3.09%
S_9	2.5%	1.4	1.62%
S_{10}	5%	1.4	1.99%

Table 2: Summary of the 10 simulations. Artificial anomalies were introduced in the data set provided by [2] with contamination levels of 2.5% or 5%. Additionally, the strength of the introduced anomalies ϵ was varied. The column "% hidden" reports the percentage of anomalies that still lie within the interquartile range of the corresponding feature.

The parameter ϵ defines how much the artificial anomalies can exceed the feature-wise maximal and minimal values. For $\epsilon < 1$ the anomalies stay within the bounds plus the 15% shift, for $\epsilon > 1$ they can be greater or smaller than the maximal or minimal value of the corresponding feature. Figure 3 in the main text shows the effect of the parameter ϵ on the strength of the placed anomalies. The first box plot corresponds to the feature **pNR2A_N** where no additional anomalies were placed. The following box plots show as black diamonds the artificial anomalies. As can be seen, with increasing ϵ the anomalies become more outlying and are more drawn away from the median of the feature.

We generated 10 simulation scenarios varying the number of introduced anomalies and their strength ϵ. We either introduced 2.5% anomalies (corresponding to 680 perturbed data points) or 5% (corresponding to 1360 perturbed data points) and also varied the strength ϵ of the introduced anomalies. The simulations are summarized in Supplemental Table 2. The column "% hidden" gives the percentage of anomalies that still lie within the interquartile range of the corresponding feature and are thus hard to detect since they represent typical values for the feature.

3.2 Handling of intrinsic anomalies

In section 3 of the main text we insert artificial anomalies in the proteomic data set provided by [2] to validate our algorithm. The data set was measured using reverse phase protein arrays (RPPA), which is a robust method for protein

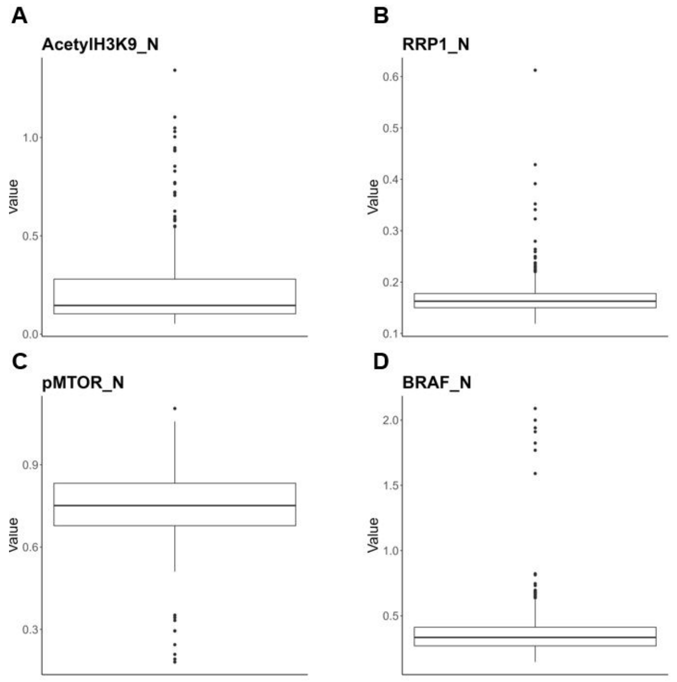

Figure 4: Boxplots of four different proteins measured by reverse phase protein arrays see [2].

quantification. Nevertheless, errors or noise in the measurements occur for example due to the limited resolution of the produced image, faulty background subtraction or saturation of the array [4], [5]. In Supplemental Figure 4 we exemplary show boxplots of four different proteins measured by RPPA from the data set. As can be seen, the measurements clearly contain some outlying data points. When we apply our algorithm to the scaled log-transformed data set without introducing artificial anomalies and set the threshold for anomaly detection according to section 2.2 in the main text, our algorithm marks 1,031 (3.79%) data points as anomalies. If we now insert the artificial anomalies, the intrinsic ones will still be detected by our algorithm, but are regarded as false positives in the calculation of the precision recall curves. Therefore, we additionally reported the PR curves for the 10 data sets described in Supplementary table 2 after excluding the 1,031 flagged anomalies from the calculation, see Figure 4B) in the main text.

3.3 Comparison to other anomaly detection methods

Search and TRace AnomalY (STRAY)

The STRAY algorithm marks a sample as an anomaly when the k-nearest neighbor distance with the maximum gap is significantly different from what

is expected when the distribution of k-nearest neighbors with the maximum gap is in the maximum domain of attraction of the Gumbel distribution [6]. For the calculation of the anomaly threshold, the authors use an approach based on extreme value theory. Since the stray algorithm aims at finding anomalous samples in a high-dimensional space instead of one-dimensional erroneous measurements within a sample, we had to apply stray for each feature separately reducing our problem to a univariate case. For the implementation we used the *find_HDoutliers* function of the R package *stray* [7]. The number k of k-nearest neighbors under consideration was chosen such that the AUC of the precision-recall curve is maximal.

Local Outlier Factor (LOF)

The LOF is an algorithm for the identification of anomalous samples by comparing the local density of a sample to the local densities of its neighbors. Points that have a significantly lower density than their neighbors are declared as anomalies or outliers. Hereby, the neighbors of a sample are determined via the k-nearest neighbors algorithm [8]. The LOF algorithms purpose is to find anomalous samples in a high-dimensional space. Therefore, we again had to submit each feature separately to obtain an anomaly score for all sample-measurement-combinations. For the implementation we used the *lof* function of the R package *dbscan* [9] and chose the number k of nearest neighbors under consideration in such a way that the AUC of the precision-recall curve was maximized.

Isolation Forest

The Isolation Forest is a model-based approach that isolates observations by randomly selecting a split value between the maximum and minimum values of a selected feature. This recursive partitioning can be represented by a tree structure and the number of splittings required to isolate a sample is equivalent to the path length from the root node to the terminating node. The anomaly score of a sample is then calculated as the average path length over a forest of such random trees [10]. The Isolation Forest uses no distance or density measures to detect anomalies but again only detects complete samples as anomalies. However, Isolation Forest can also be applied to mixed data where categorical variables are included using one hot encoding. In our analyses we made use of this by supplying not each continuous feature separately but also including the categorical information given by the three discrete features genotype, treatment and behavior. Therefore, the anomaly scores for each feature were obtained by an Isolation Forest model trained on four variables: the three discrete features and the corresponding continuous feature. In the following analyses we used the *sklearn.ensemble.IsolationForest* function as offered by the Python module scikit-learn [11].

We compared ADMIRE to the three anomaly detection methods on the log-transformed simulations (Supplemental Figure 5) and the log-transformed simulations after correcting for the 1,031 intrinsic anomalies identified by ADMIRE (Supplemental Figure 6). The precision-recall curves and AUCs for the three algorithms can directly be compared to the respective precision-recall curves

and AUCs of ADMIRE (Figure 4, main text). As can be seen, ADMIRE outperforms all algorithms in all simulations. This is also true after correcting for the intrinsic anomalies. The differences in the AUCs are especially prominent for the simulations with small ϵ, corresponding to less outliers and more hidden anomalies. Here, the information given by the categorical variables becomes crucial in the anomaly detection, as exemplified by the AUCs of the Isolation Forest. We ran the Isolation Forest for each simulation twice: first without including the categorical variables (Supplemental Figures 5 C and 6 C) and again also supplying the discrete features (Figures 5 D and 6 D). The increasing AUCs demonstrate the necessity of performing anomaly detection with respect to the phenotypical information.

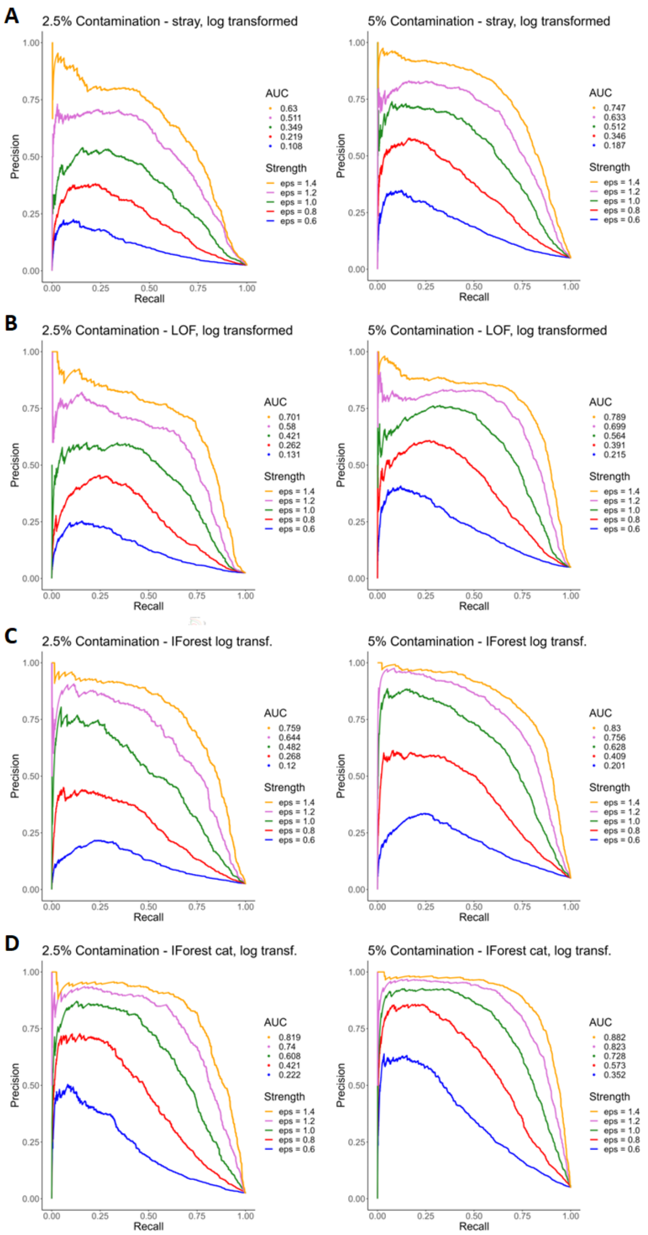

Figure 5: Precision-Recall curves for the log-transf. and standardized simulations with 2.5% and 5% contamination:
A) Performance of stray
B) Performance of LOF
C) Performance of Isolation Forest applied only to continuous data
D) Performance of Isolation Forest applied to continuous and categorical data

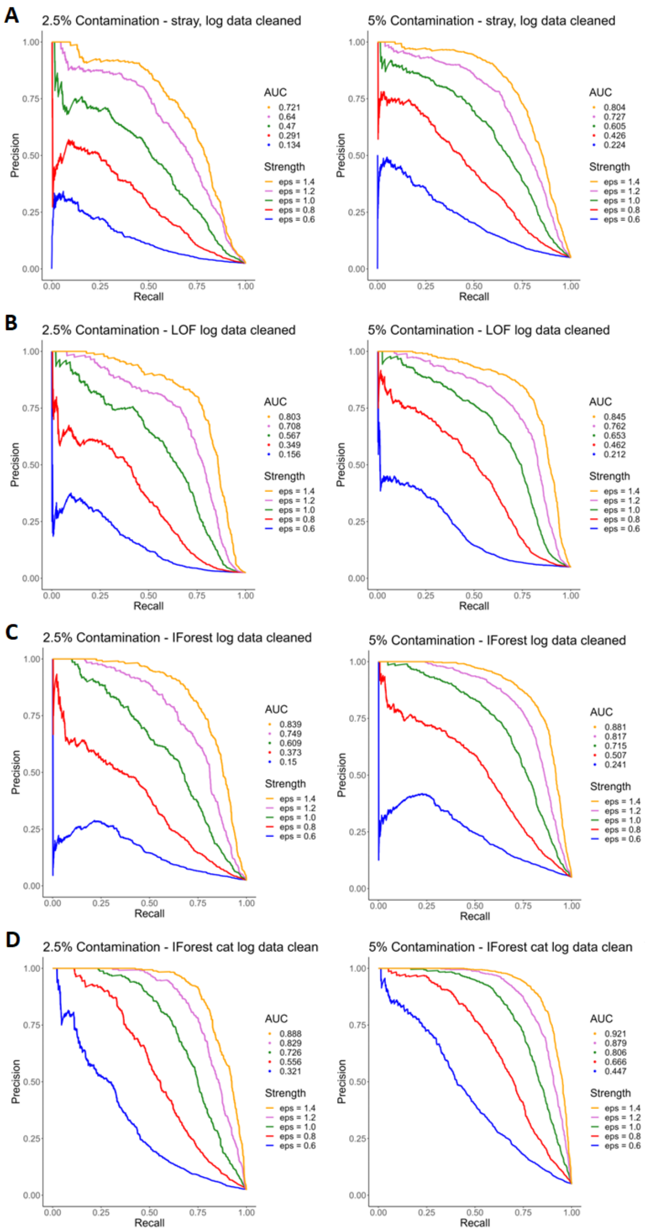

Figure 6: Precision-Recall curves for the log-transf. and standardized simulations with 2.5% and 5% contamination after correcting for intrinsic anomalies:
A) Performance of stray
B) Performance of LOF
C) Performance of Isolation Forest applied only to continuous data
D) Performance of Isolation Forest applied to continuous and categorical data

4 Supplementary information for the validation of ADMIRE on the metabolic data set

4.1 Experimental setup

Cell culture experiments of P493/6 cells
P493/6 B-cells were cultured in RPMI 1640 media (Lonza, Basel, Switzerland) supplemented with 11.1 mM glucose, Penicillin-Streptomycin-L-Glutamine (PSG) and 10% fetal calf serum (FCS) (Gibco / Thermo Fisher Scientific, Waltham, MA, USA) in a humidified atmosphere with 5% CO_2 at 37 °C. MYC transcription was repressed by adding 1 ng/mL doxycycline for 24 h at a cell density of 1 x 10^6 cells/mL (MYC^{low}). Without the addition of doxycycline the inducable MYC transcript was not suppressed any longer and MYC^{high} cells were obtained. Cell counting was performed using a hemocytometer and cell viability was determined by the trypan blue exclusion test of cell viability. Cells were thawed and continuously split for one week. All experiments were performed in the absence of the EBNA2 latency III proliferation program (without estrogen addition). Subsequently, cells were washed once with 15 mL PBS and seeded at a count of 1 x 10^6 cells/mL in fresh medium. As a control, cell culture flasks containing medium only were incubated in parallel. After 24 h of cultivation, cells were counted and viability was monitored. Only samples with >90% vitality were used for metabolomic analyses. Cell suspensions were centrifuged at 120 x g for 7 min at 4 °C, supernatants were collected and cell pellets were washed once with 10 mL of cold PBS. The samples were stored at -80 °C for later metabolomic analyses.

Stimulation experiments
For the stimulation experiments MYC^{low} or MYC^{high} cells were treated with single doses or combinations of 130 ng/mL α-IgM $F(ab)_2$ fragments, 100 ng/mL sCD40L (Autogen Bioclear, Wiltshire, UK), 100 ng/mL rhIGF-1 (Peprotech, Hamburg, Germany), 25 ng/mL IL-10 (Peprotech) and/or 0.5 µM CpG (Invivogen, San Diego, USA) for 24 h at 1 x 10^6 cells/mL. The experimental design is given in Supplemental Figure 7.

Quantification of metabolites in cell supernatants
A complete list of all analyzed metabolites including the employed analytical methods is given in Supplemental Table 3. As detailed below raw metabolite concentrations were determined either by NMR spectroscopy or mass spectrometry. To determine the amount of secreted or consumed metabolites the respective medium controls were subtracted from the raw concentration values followed by a normalization to the area under the corresponding growth curve to take differences in cell growth into account.

NMR analyses of cell culture supernatants
For protein removal ultrafiltration was performed with all specimens. To that

Nr.	Batch	CD40L	α-IGM	IGF-1	CpG	IL-10	MYC	Nr.	Batch	CD40L	α-IGM	IGF-1	CpG	IL-10	MYC
1	1	1	0	0	0	0	high	51	6	1	0	0	0	0	high
2	1	0	1	0	0	0	low	52	6	0	1	0	0	0	low
3	1	0	0	0	0	0	low	53	6	0	0	0	0	0	low
4	1	0	0	0	0	0	high	54	6	0	0	0	0	0	high
5	1	0	1	1	1	1	high	55	6	0	0	0	0.2	1	high
6	1	1	1	1	1	1	high	56	6	0.2	0.2	0.2	1	1	high
7	1	1	1	0	1	0	high	57	6	0.2	1	1	0	0	high
8	1	1	0	0	1	1	low	58	6	1	1	0	1	1	low
9	1	0.2	1	1	0	1	low	59	6	1	1	1	1	1	low
10	1	0	0	0	1	1	low	60	6	0.2	0	1	0.2	1	low
11	2	0	1	0	0	0	high	61	7	0	1	0	0	0	high
12	2	0	0	1	0	0	low	62	7	0	0	1	0	0	low
13	2	0	0	0	0	0	low	63	7	0	0	0	0	0	low
14	2	0	0	0	0	0	high	64	7	0	0	0	0	0	high
15	2	0	1	0.2	0.2	0	high	65	7	0.2	1	1	0	1	high
16	2	1	0.2	0.2	0	0	high	66	7	1	0.2	0	0	1	high
17	2	1	1	1	1	0.2	high	67	7	1	1	0	1	1	high
18	2	0.2	0.2	0.2	0.2	0.2	low	68	7	0	0.2	0	0.2	1	low
19	2	1	0.2	0	0	1	low	69	7	1	1	1	1	0.2	low
20	2	0.2	1	0.2	0	0.2	low	70	7	1	1	0	1	0	low
21	3	0	0	1	0	0	high	71	8	0	0	1	0	0	high
22	3	0	0	0	1	0	low	72	8	0	0	0	1	0	low
23	3	0	0	0	0	0	low	73	8	0	0	0	0	0	low
24	3	0	0	0	0	0	high	74	8	0	0	0	0	0	high
25	3	0.2	0.2	0.2	0	0	high	75	8	0	0.2	0	0.2	1	high
26	3	0.2	1	0	0	1	high	76	8	0.2	0.2	0.2	0.2	0.2	high
27	3	1	0	0	1	1	high	77	8	0.2	1	0.2	0	0.2	high
28	3	0.2	1	0.2	1	0	low	78	8	0	1	1	1	1	low
29	3	0	0	0	0.2	1	low	79	8	0.2	0.2	0	1	1	low
30	3	0.2	1	0.2	0.2	0.2	low	80	8	0.2	0.2	0.2	1	1	low
31	4	0	0	0	1	0	high	81	9	0	0	0	1	0	high
32	4	0	0	0	0	1	low	82	9	0	0	0	0	1	low
33	4	0	0	0	0	0	low	83	9	0	0	0	0	0	low
34	4	0	0	0	0	0	high	84	9	0	0	0	0	0	high
35	4	0.2	1	0.2	0.2	0.2	high	85	9	0.2	1	1	0.2	0	high
36	4	1	1	0.2	0.2	0	high	86	9	0.2	0.2	1	0	0.2	high
37	4	0.2	0	1	0.2	0	high	87	9	1	0	1	1	0.2	high
38	4	1	1	0.2	0.2	0	low	88	9	0	1	0.2	0.2	0	low
39	4	0.2	1	1	0.2	0	low	89	9	0.2	0.2	1	0	0.2	low
40	4	1	0	1	1	0.2	low	90	9	1	0	1	0.2	0	low
41	5	0	0	0	0	1	high	91	10	0	0	0	0	1	high
42	5	1	0	0	0	0	low	92	10	1	0	0	0	0	low
43	5	0	0	0	0	0	low	93	10	0	0	0	0	0	low
44	5	0	0	0	0	0	high	94	10	0	0	0	0	0	high
45	5	0	0	0	1	1	high	95	10	1	0	1	0.2	0	high
46	5	0.2	0.2	0	1	1	high	96	10	0.2	1	0.2	1	0	high
47	5	0.2	0	0.2	1	0	high	97	10	0.2	0	1	0.2	1	high
48	5	0.2	0.2	0.2	0	0	low	98	10	0.2	1	1	0	0	low
49	5	0.2	1	0	0	1	low	99	10	0.2	0	1	0.2	0	low
50	5	0.2	0	0.2	1	0	low	100	10	1	0.2	0.2	0	0	low

Figure 7: Matrix of stimuli combinations used for metabolomic investigations. P493/6 cells under MYC^{high} or MYC^{low} condition were treated with single doses or combinations of α-IgM, CD40L, CpG, IGF-1 or IL-10. Experiments were performed in 10 batches, each containing an unstimulated MYC^{high} (highlighted in orange) and MYC^{low} (highlighted in grey) ctrl sample. Concentration 1 = full dosis of stimulation (130 ng/mL α-IgM, 100 ng/mL CD40L, 0.5 µM CpG, 100 ng/mL IGF-1, 25 ng/mL IL-10), concentration 0.2 = reduced concentration (26 ng/mL α-IgM, 20 ng/mL CD40L, 0.1 µM CpG, 20 ng/mL IGF1, 5 ng/mL IL-10), concentration 0 = no stimuli added.

end, centrifugal filters were prewashed with 3 mL H_2O (4,000 x g, 30 min, room temperature) prior to ultrafiltration of 1.5 mL of supernatant (2,000 x g, 60 min, 4 °C). 400 µL of the ultrafiltrate were mixed with 200 µL of 0.1 mol/L phosphate buffer, pH 7.4, and 50 µL of 0.75% (w) 3 trimethylsilyl 2,2,3,3 tetradeuteropropionate (TSP) dissolved in deuterium oxide as internal standard (Sigma Aldrich). NMR analyses were performed on a 600 MHz Avance III spectrometer (BrukerBioSpin, Rheinstetten, Germany). For every specimen 1D ^{1}H spectra were acquired following established protocols [12]. In short, 1D NOESY (nuclear overhauser enhancement spectroscopy) pulse sequences with presaturation during relaxation and mixing time together with additional spoil gradients for water suppression were used. 128 scans were collected into 64k data points over a 20 ppm spectral width using a relaxation delay of 4 s, an acquisition time of 2.66 s and a mixing time of 0.01 s. Absolute metabolite concentrations were determined with Chenomx 9.02 (Chenomx Inc.Edmonton, Canada).

Quantification of amino acids by mass spectrometry
Free amino acids in cell culture supernatants were derivatized with propylchloroformate/propanol and analyzed by reversed-phase HPLC-MS/MS as described previously [13]. Briefly, 10 µL of stable isotope labeled internal standard containing [U-^{13}C, U-^{15}N] amino acid mix dissolved in water were added to the evaporated supernatants before bringing the final volume to 200 µL. The internal standard mix was prepared based on a cell free amino acid mix obtained from Eurisotop, Saint Aubin, France (IS 1). To this mix asparagine, glutamine, hippurate, ornithine, and tryptophane were manually added (IS 2), as they were not included in the commercial mix. The mass spectrometer was operated in multi-reaction-monitoring (MRM) mode using one transition for each analyte and its corresponding internal standard. Quantification was performed using calibration curves based on the chromatographic peak area ratio (analyte/internal standard) and the corresponding nominal concentration ratio (analyte/internal standard).

Quantification of intermediates of the methionine and polyamine metabolism by mass spectrometry
Intermediates of the methionine and polyamine metabolism in cell culture supernatants were determined as described previously ([14]). Shortly, 10 µL of stable isotope labeled internal standard containing 1 µM [1′,2′,3′,4′,5′-$^{13}C_5$]-methylthioadenosine, 1 µM [1′,2′,3′,4′,5′-$^{13}C_5$]-adenosine, 5 µM d_7-ornithine, 5 µM d_3-S-adenosyl methionine, 5 µM U $^{13}C_5$-adenine, 10 µM $^{15}N_3$-spermidine, 10 µM U $^{13}C_4$-putrescine, 10 µM d_8-spermine and additional 10 µL of 10 µM d_4-homocysteine in aqueous 0.1 M acetic acid had been added to the evaporated cell extracts before reconstituting in 100 µL of aqueous 0.1 M acetic acid. For analysis of cell culture supernatants 10 µL of medium were extracted with 150 µL MeOH/1 M acetic acid 80:20, v/v, two times and reconstituted in 100 µL of 0.1 M acetic acid after evaporation.

Quantification of tryptophan metabolites by mass spectrometry

Tryptophan and its key metabolites were quantified in cell culture supernatants and cell pellets as described previously [15]. Shortly, 10 μL of stable isotope labeled internal standard containing $^{2}H_4$-melatonin and $^{2}H_2$-indoleacetic-acid-Gly (1 μM each); $^{2}H_4$-anthranilic acid, $^{2}H_4$-tryptamine, and $^{2}H_4$-serotonine (2 μM each); $^{2}H_4$-nicotinic acid, $^{2}H_4$-nicotinamid, isatin, and $^{2}H_5$-hydroxy-indoleacetic acid (10 μM each), as well as 50 mg dry weight yeast extract per milliliter containing $^{13}C_{10}$-kynurenine, $^{13}C_7$-3-hydroxyanthranilic acid, $^{13}C_{11}$-tryptophan, $^{13}C_{10}$-xanthurenic acid, $^{13}C_{10}$-kynurenic acid, $^{13}C_7$-quinolinic acid, $^{13}C_{10}$-3-hydroxykynurenine, and $^{13}C_{10}$-indole-3-acetic acid in water had been added to the evaporated cell extracts before reconstituting in 100 μL of 0.1 % formic acid in water. For the preparation of cell culture supernatant, 10 μL of the internal standard mix was added to 10 μL of MeOH extracted (80:20, v/v) supernatant and afterwards diluted to 100 μL with 0.1 % formic acid in water.

Generation of final metabolite table

To assess the amount of metabolites consumed from or released to the cell-culture medium, the respective values of the medium controls i.e. the samples that were never in contact with the cells were subtracted from the values of the samples with cells. This results in negative values for metabolites that were consumed from the medium and positive values for metabolites that were released to the medium. Therefore, the final metabolite table contains both negativ and positiv concentration values.

**metabolite**	**general method**	**specific method**
alanine	MS	amino acid method IS 1
arginine	NMR	NMR method
asparagine	MS	amino acid method IS 2
aspartate	MS	amino acid method IS 1
cystine	MS	amino acid method IS 1
glutamine	MS	amino acid method IS 2
glutamate	MS	amino acid method IS 1
glycine	MS	amino acid method IS 1
hippuric acid	MS	amino acid method IS 2
histidine	MS	amino acid method IS 1
isoleucine	MS	amino acid method IS 1
leucine	MS	amino acid method IS 1
lysine	MS	amino acid method IS 1
methionine	MS	amino acid method IS 1
ornithine	MS	amino acid method IS 2
phenylalanine	MS	amino acid method IS 1
proline	MS	amino acid method IS 1
serine	MS	amino acid method IS 1
threonine	MS	amino acid method IS 1
tryptophan	MS	amino acid method IS 2
tyrosine	MS	amino acid method IS 1
valine	MS	amino acid method IS 1
D-glucose	NMR	NMR method
lactic acid	NMR	NMR method
acetone	NMR	NMR method
formic acid	NMR	NMR method
pyruvic acid	NMR	NMR method
succinic acid	NMR	NMR method
2-oxoisocaproic acid	NMR	NMR method
choline	NMR	NMR method
4-hydroxyproline	NMR	NMR method
pyroglutamic acid	NMR	NMR method
Anthranilic acid	MS	tryptophan method
3-hydroxyanthranilic acid	MS	tryptophan method
5-hydroxy-indole-acetic acid	MS	tryptophan method
3-hydroxykynurenine	MS	tryptophan method
indole-3-acetic acid	MS	tryptophan method
indole-lactic acid	MS	tryptophan method
kynurenic acid	MS	tryptophan method
kynurenine	MS	tryptophan method
nicotinic acid	MS	tryptophan method
nicotin amid	MS	tryptophan method
serotonine	MS	tryptophan method
adenosine	MS	methionine method
5′-methylthioadenosine	MS	methionine method
ornithine	MS	methionine method
putrescine	MS	methionine method
S-adenosylmethionine	MS	methionine method
spermidine	MS	methionine method

Table 3: Quantified metabolites

4.2 Detection of artificial discrete artefacts

A

Rank	Sample	Feature	Observed	Random
1	9	Batch	1.781	5.107
2	12	Batch	1.682	4.569
3	4	Batch	1.624	4.292
4	3	Batch	1.617	4.077
5	56	Batch	1.491	3.926
6	1	Batch	1.378	3.754
7	31	Batch	1.376	3.629
8	8	Batch	1.368	3.495
9	10	Batch	1.356	3.388
10	2	Batch	1.299	3.297

B

Rank	Sample	Feature	Observed	Random
1	86	MYC	5.694	5.100
2	53	MYC	5.201	4.492
3	9	Batch	1.781	4.171
4	12	Batch	1.683	3.965
5	4	Batch	1.630	3.813
6	3	Batch	1.616	3.670
7	56	Batch	1.524	3.550
8	1	Batch	1.382	3.442
9	31	Batch	1.377	3.345
10	8	Batch	1.366	3.270

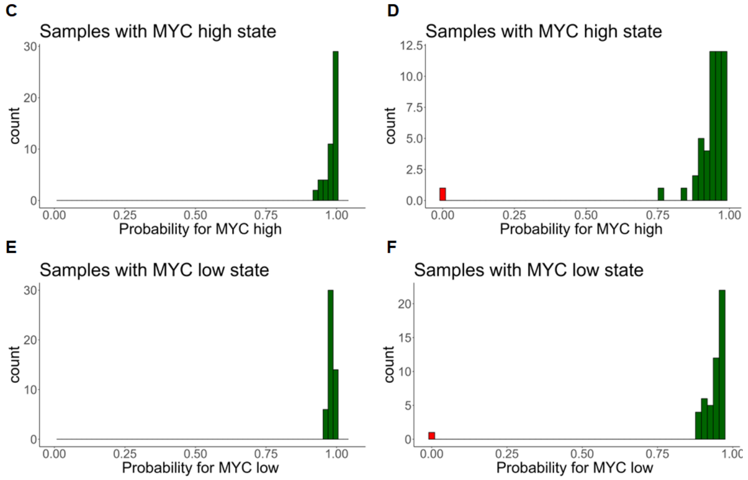

Figure 8: A) 10 highest ranking observed and random discrete scores without artificially introduced anomalies. The threshold is marked in green.
B) 10 highest ranking observed and random discrete scores with artficially introduced anomalies (red rows). The threshold is marked in green.
C) Distribution of estimated probabilities for samples with MYC state ”high” without artificial anomalies.
D) Distribution of estimated probabilities for samples with MYC state ”high” with artificially introduced anomaly (corresponding probability marked in red).
E) Distribution of estimated probabilities for samples with MYC state ”low” without artificial anomalies.
F) Distribution of estimated probabilities for samples with MYC state ”low” with artificially introduced anomaly (corresponding probability marked in red).

When ADMIRE is applied to the metabolic data set, it detects no discrete anomalies suggesting that all discrete features are correct (see Supplemental Figure 8A). To further validate the detection of discrete anomalies we artificially changed the MYC state in two samples and reapplied ADMIRE. Now, ADMIRE detects two anomalies (Supplemental Figure 8B), which correspond to the artificially introduced ones. This is also reflected by the distribution of the estimated probabilities for the corresponding MYC state without artificial

anomalies (Supplemental Figures 8 C and E) and with artificial anomalies (Supplemental Figures 8 D and F). The probabilities of the artificial anomalies are marked in red.

4.3 Distribution of detected continuous anomalies in samples

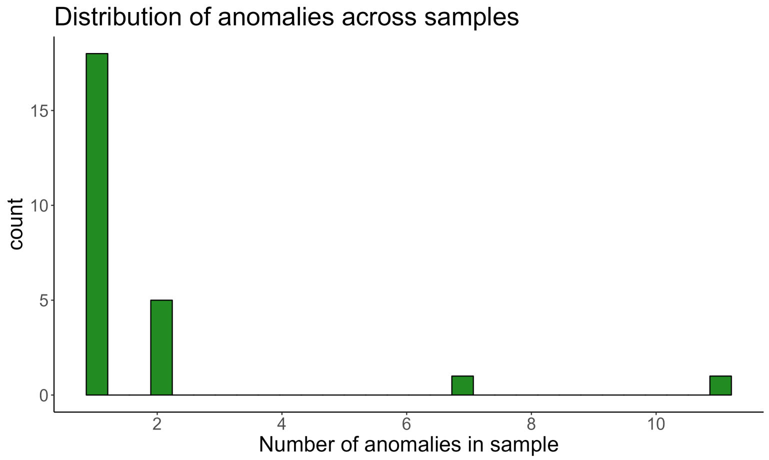

Figure 9: Distribution of the number of detected continuous anomalies per sample in the metabolic data set.

ADMIRE detects 46 continuous anomalies in the metabolic data set. Most samples do not contain any detected anomalies at all (75% of the samples). There are two remarkable exceptions, see Supplemental Figure 9. Sample 92 contains seven detected anomalies and sample 7 contains 11 detected anomalies.

5 Performance dependency on contamination rate

So far, in our simulation up to 5% of anomalies were included. Here, we investigate how ADMIRE behaves when the data contains considerable more noise. We performed additional simulations where we used algorithm 1 and introduced up to 70% of artificial anomalies in the data set from [2]. The strength of the anomalies was set to $\epsilon = 1.0$. In Figure 10 we show for each simulation the number of artificially introduced anomalies (black), the number of anomalies ADMIRE detects (red) and the true positive anomalies amongst them (green). Until up to 45% of introduced anomalies the performance of ADMIRE stays stable with an equal ratio between detected and true positive anomalies. This is also supported by the balanced accuracy ((sensitivity + specificity)/2) which we reported in Supplemental Table 4). From 50% of introduced anomalies

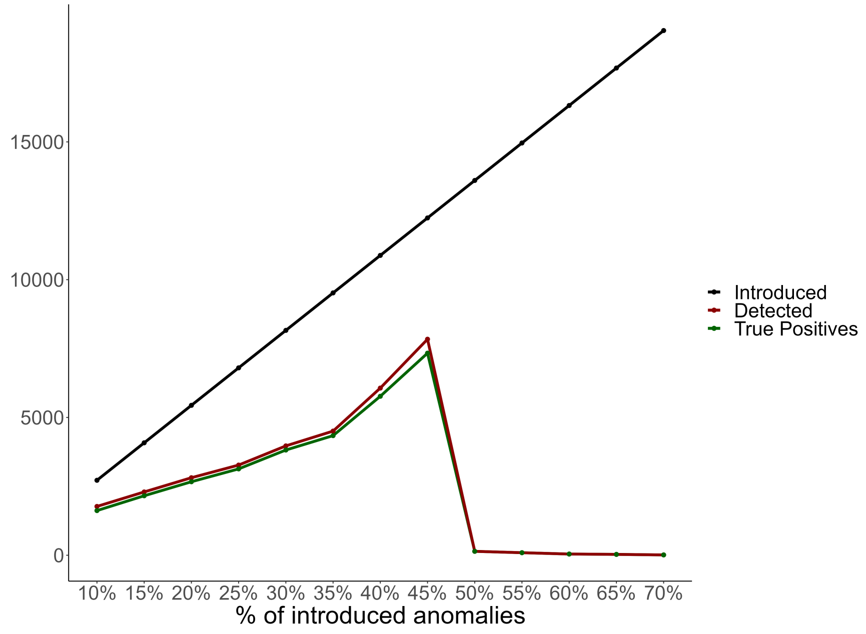

Figure 10: Dependency of the anomaly detection on the contamination rate. Simulation where we placed between 10% and 70% artificial anomalies in a data set. The black curve shows the number of artificial anomalies, red corresponds to the number of anomalies detected by ADMIRE and green to the true positive anomalies amongst the detected.

on however, ADMIRE breaks down and is not able to infer the data structure anymore. The number of detected anomalies goes down to almost zero (for 70% only 12 anomalies are detected). This is not surprising since for 50% of introduced anomalies, ADMIRE tries to model the predominating noise in the data set instead of the masked, true structure.

Simulation	Balanced Accuracy
10%	0.795
15%	0.761
20%	0.742
25%	0.727
30%	0.73
35%	0.723
40%	0.756
45%	0.783
50%	0.505
55%	0.503
60%	0.501
65%	0.501
70%	0.5

Table 4: Balanced accuracy of ADMIRE's anomaly detection for increasing amount of artificially introduced anomalies.

6 Data distribution

ADMIRE assumes that the conditional distribution of a discrete variable given all other variables is multinomial with probabilitis given by muticlass logistic regressions. The continuous variables are assumed to follow conditional normal distributions with the means given by linear regressions on the respective continuous features. However, when we compare the data from [2] with the normal distribution (Supplemental Figure 11A) we observe that our data used to generate the simulations is in fact not normally distributed but follows some distribution with higher kurtosis. The same holds true for the conditional distributions of the data set. In Supplemental Figure 11B) we show the normal QQ plots of the residuals obtained by estimating the raw data set (without any artificial anomalies) using ADMIRE (through equation (3) from the main text). Also, as can be seen in Supplemental Figures 11C) and D) the data set and the residuals are not log-normally distributed either.

The distribution of the metabolomic data set does not have such a high kurtosis as the proteomic data set from [2] but still has heavy tails compared to the normal distribution (see Supplemental Figure 12A). When we look at the conditional distributions through the residuals obtained by ADMIRE's estimates we can also detect non-normal behaviour.

We conclude that even though ADMIRE assumes conditional normality of the features it still performs well if these assumptions are not met.

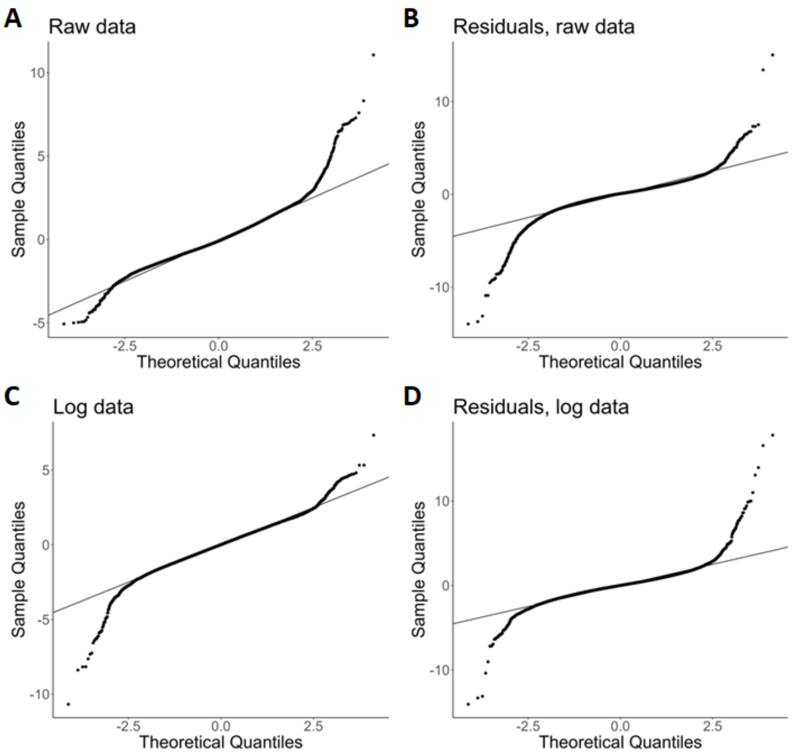

Figure 11: A) Normal QQ-Plot of the raw data from [2].
B) Normal QQ-plot of the residuals, where ADMIRE was trained on the raw data.
C) Normal QQ-Plot of the log-transformed data from [2].
D) Normal QQ-plot of the residuals, where ADMIRE was trained on the log-transformed data.

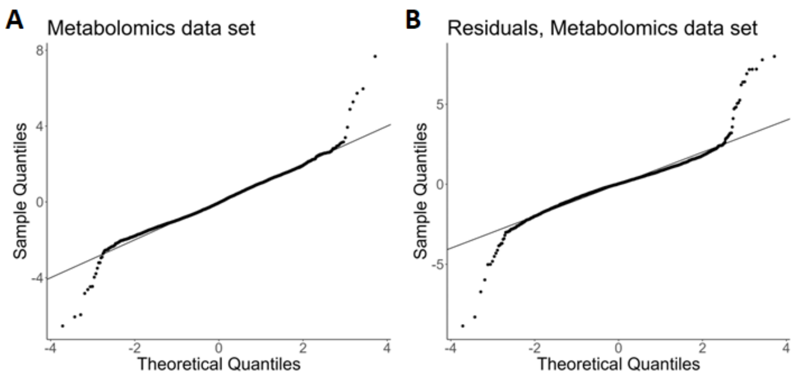

Figure 12: A) Normal QQ-Plot of the metabolomic data set.
B) Normal QQ-plot of the residuals from the metabolomic data set.

7 Runtime analysis

The runtime of ADMIRE is mostly driven by the fitting of the Mixed Graphical Models. In each run of ADMIRE the parameter set $\Theta = \{\{\beta_{js}\}, \{\alpha_j\}, \{\rho_{jt}\}, \{\phi_{rt}\}, j, s \in \{1 \ldots p\}, r, t \in \{1 \ldots q\}\}$ has to be estimated which is computational costly. More precisely let p be the number of continuous features and q the number of discrete features in the model. Since β_{js} and ϕ_{sj} are symmetric we have to estimate $\frac{p^2}{2} + pq + \frac{q^2}{2} = \frac{1}{2}(p+q)^2$ parameters resulting in a quadratic runtime of $O((p+q)^2)$, see also reference [16]. To assess the runtime more closely we used a published proteomics data set from reference ([17]), which consists of 84 samples and 780 proteins and measured the time ADMIRE takes to perform one complete step of the leave-one-out cross validation (for one fixed λ all 84 samples of the data set are estimated by the leave-one-out approach described in the main text). To outline the runtime dependency on the number of features we iteratively increased the number of features in the data set supplied to ADMIRE. The results are summarized in Supplemental Figure 13, where we report for each employed number of features the run-time of ADMIRE in seconds. The runtime analysis was performed on a Dell PowerEdge R750 Server with 2 Intel Xeon CPUs (28C/56T) running Ubuntu 22.04.1 LTS as operating system.

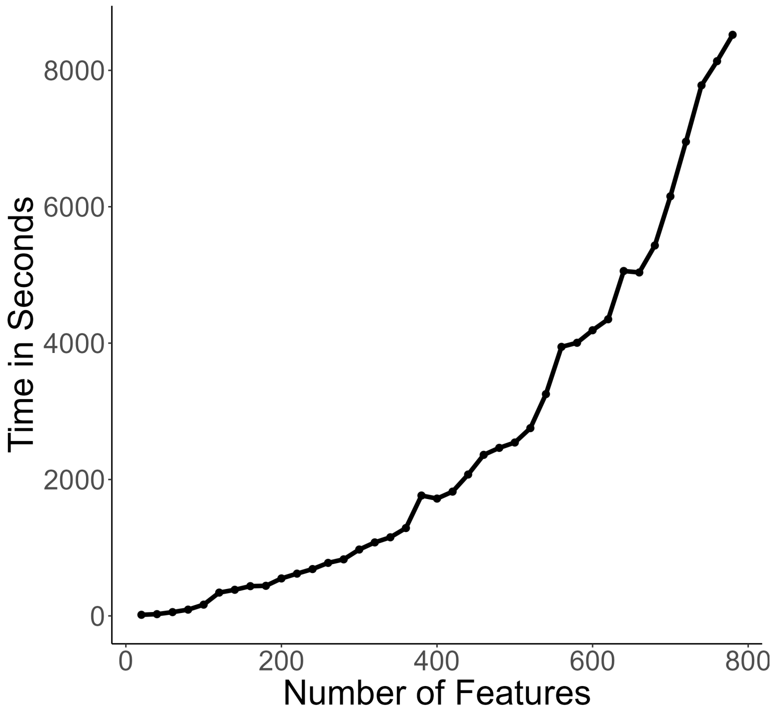

Figure 13: Runtime dependency on the number of features included in the data set. Each dot in the graph corresponds to the time in seconds ADMIRE takes to perform one complete step of the leave-one-out cross validation.

8 Visualization of Mixed Graphical Models

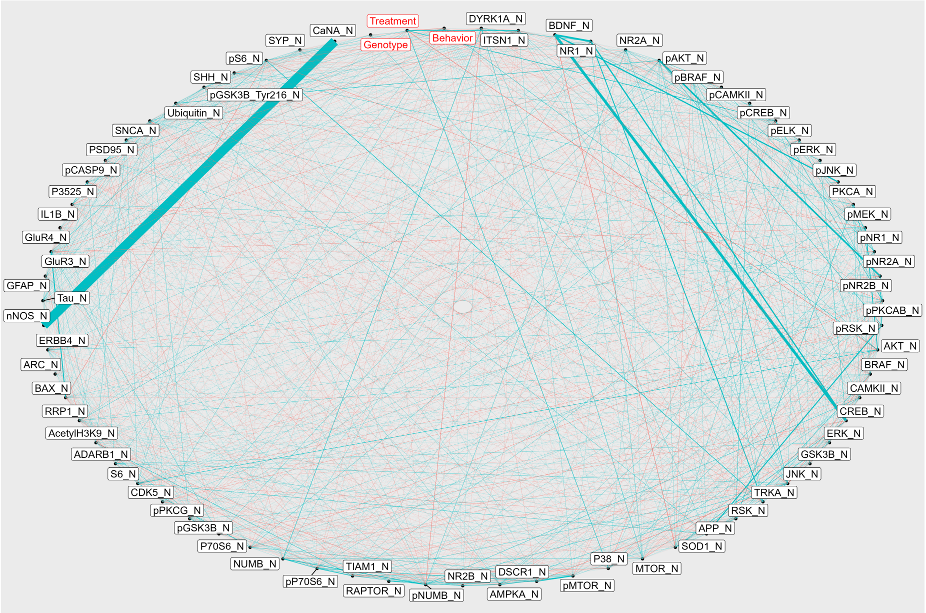

Figure 14: Visualization of the fitted MGM for the RPPA data set [2]. Blue edges represent positive relations, red edges negative relations. The thickness of the edge corresponds to the value of the edge weight.

In Supplemental Figure 14 the graph structure for the RPPA data set ([2]) according to the estimated parameter set Θ was plotted. In red the discrete variables Genotype, Treatment and Behavior are shown with respect to the reference level. The edges connecting the nodes represent the conditional dependency structure of the variables. Hereby, red edges denote a negative correlation, blue edges a positive correlation and the thickness reflects the value of the edge weight. Supplemental Figure 15 shows the corresponding plot for the metabolic data set.

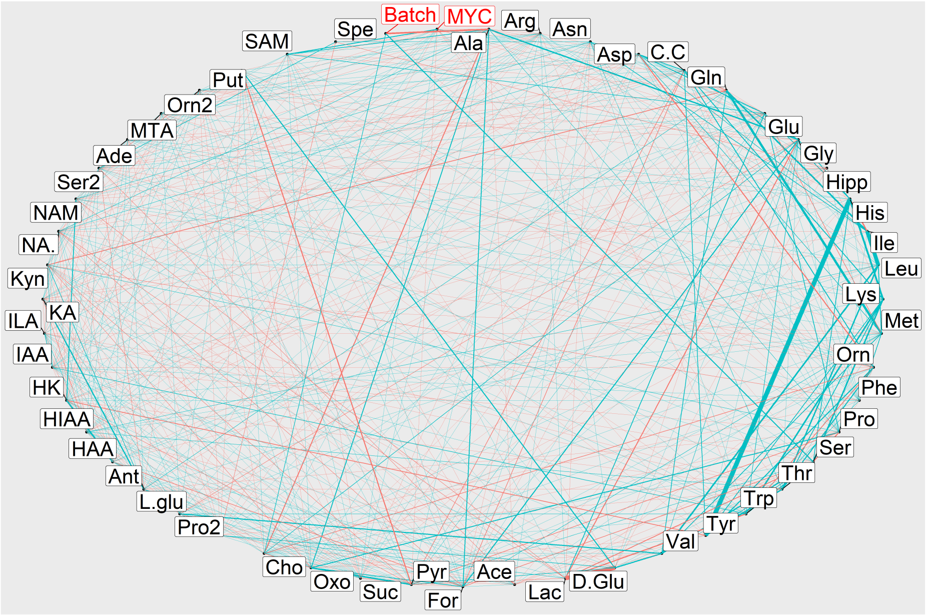

Figure 15: Visualization of the fitted MGM for the metabolomic data set. Blue edges represent positive relations, red edges negative relations. The thickness of the edge corresponds to the value of the edge weight.

References

[1] Daniel J. Stekhoven and Peter Bühlmann. "MissForest—non-parametric missing value imputation for mixed-type data". In: *Bioinformatics* 28.1 (Oct. 2011), pp. 112–118. ISSN: 1367-4803. DOI: `10.1093/bioinformatics/btr597`. URL: `https://doi.org/10.1093/bioinformatics/btr597`.

[2] Clara Higuera, Katheleen J. Gardiner, and Krzysztof J. Cios. "Self-organizing feature maps identify proteins critical to learning in a mouse model of down syndrome". In: *PLOS ONE* 10.6 (June 2015). DOI: `10.1371/journal.pone.0129126`.

[3] Georg Steinbuss and Klemens Böhm. "Hiding outliers in high-dimensional data spaces". In: *International Journal of Data Science and Analytics* 4.3 (Sept. 2017), pp. 173–189. DOI: `10.1007/s41060-017-0068-8`.

[4] Li Zhang et al. "Serial dilution curve: a new method for analysis of reverse phase protein array data". In: *Bioinformatics* 25.5 (Jan. 2009), pp. 650–654. ISSN: 1367-4803. DOI: `10.1093/bioinformatics/btn663`. eprint: `https://academic.oup.com/bioinformatics/article-pdf/25/5/650/442801/btn663.pdf`.

[5] Huma Shehwana et al. "RPPA SPACE: an R package for normalization and quantitation of Reverse-Phase Protein Array data". In: *Bioinformatics* (Oct. 2022). btac665. ISSN: 1367-4803. DOI: 10.1093/bioinformatics/btac665. eprint: https://academic.oup.com/bioinformatics/advance-article-pdf/doi/10.1093/bioinformatics/btac665/46560331/btac665.pdf.

[6] Priyanga Dilini Talagala, Rob J. Hyndman, and Kate Smith-Miles. "Anomaly Detection in High-Dimensional Data". In: *Journal of Computational and Graphical Statistics* 30.2 (2021), pp. 360–374. DOI: 10.1080/10618600.2020.1807997. URL: https://doi.org/10.1080/10618600.2020.1807997.

[7] Priyanga Dilini Talagala. *stray: Anomaly Detection in High Dimensional and Temporal Data*. R package version 0.1.1. 2020. URL: https://CRAN.R-project.org/package=stray.

[8] Markus M. Breunig et al. "LOF". In: *ACM SIGMOD Record* 29.2 (2000), pp. 93–104. DOI: 10.1145/335191.335388.

[9] Michael Hahsler, Matthew Piekenbrock, and Derek Doran. "dbscan: Fast Density-Based Clustering with R". In: *Journal of Statistical Software* 91.1 (2019), pp. 1–30. DOI: 10.18637/jss.v091.i01. URL: https://www.jstatsoft.org/index.php/jss/article/view/v091i01.

[10] Fei Tony Liu, Kai Ming Ting, and Zhi-Hua Zhou. "Isolation Forest". In: *2008 Eighth IEEE International Conference on Data Mining*. 2008, pp. 413–422. DOI: 10.1109/ICDM.2008.17.

[11] F. Pedregosa et al. "Scikit-learn: Machine Learning in Python". In: *Journal of Machine Learning Research* 12 (2011), pp. 2825–2830.

[12] Wolfram Gronwald et al. "Urinary metabolite quantification employing 2D NMR spectroscopy". In: *Analytical Chemistry* 80.23 (2008), pp. 9288–9297. DOI: 10.1021/ac801627c.

[13] Annemieke T. van der Goot et al. "Delaying aging and the aging-associated decline in protein homeostasis by inhibition of tryptophan degradation". In: *Proceedings of the National Academy of Sciences* 109.37 (2012), pp. 14912–14917. DOI: 10.1073/pnas.1203083109.

[14] Axel P. Stevens et al. "Quantification of intermediates of the methionine and polyamine metabolism by liquid chromatography–tandem mass spectrometry in cultured tumor cells and liver biopsies". In: *Journal of Chromatography A* 1217.19 (2010), pp. 3282–3288. DOI: 10.1016/j.chroma.2010.01.025.

[15] Wentao Zhu et al. "Quantitative profiling of tryptophan metabolites in serum, urine, and cell culture supernatants by liquid chromatography–tandem mass spectrometry". In: *Analytical and Bioanalytical Chemistry* 401.10 (2011), pp. 3249–3261. DOI: 10.1007/s00216-011-5436-y.

[16] Jason D. Lee and Trevor J. Hastie. "Learning the Structure of Mixed Graphical Models". In: *Journal of Computational and Graphical Statistics* 24.1 (2015), pp. 230–253. DOI: 10.1080/10618600.2014.900500.

[17] Jörg Reinders et al. "Platform independent protein-based cell-of-origin subtyping of diffuse large B-cell lymphoma in formalin-fixed paraffin-embedded tissue". In: *Scientific Reports* 10.1 (2020). DOI: 10.1038/s41598-020-64212-z.
